# Impact of clinical phenotypes on management and outcomes in European atrial fibrillation patients: a report from the ESC-EHRA EURObservational Research Programme in AF (EORP-AF) General Long-Term Registry

**DOI:** 10.1186/s12916-021-02120-3

**Published:** 2021-10-20

**Authors:** Marco Proietti, Marco Vitolo, Stephanie L. Harrison, Deirdre A. Lane, Laurent Fauchier, Francisco Marin, Michael Nabauer, Tatjana S. Potpara, Gheorghe-Andrei Dan, Giuseppe Boriani, Gregory Y. H. Lip, on behalf of the ESC-EHRA EORP-AF Long-Term General Registry Investigators, G. Boriani (Chair), G. Y. H. Lip, L. Tavazzi, A. P. Maggioni, G.-A. Dan, T. Potpara, M. Nabauer, F. Marin, Z. Kalarus, L. Fauchier, R. Ferrari, A. Shantsila, A. Goda, G. Mairesse, T. Shalganov, L. Antoniades, M. Taborsky, S. Riahi, P. Muda, I. García Bolao, O. Piot, M. Nabauer, K. Etsadashvili, E. N. Simantirakis, M. Haim, A. Azhari, J. Najafian, M. Santini, E. Mirrakhimov, K. Kulzida, A. Erglis, L. Poposka, M. R. Burg, H. Crijns, Ö. Erküner, D. Atar, R. Lenarczyk, M. Martins Oliveira, D. Shah, G.-A. Dan, E. Serdechnaya, T. Potpara, E. Diker, G. Y. H. Lip, D. Lane, E. Zëra, U. Ekmekçiu, V. Paparisto, M. Tase, H. Gjergo, J. Dragoti, A. Goda, M. Ciutea, N. Ahadi, Z. el Husseini, M. Raepers, J. Leroy, P. Haushan, A. Jourdan, C. Lepiece, L. Desteghe, J. Vijgen, P. Koopman, G. Van Genechten, H. Heidbuchel, T. Boussy, M. De Coninck, H. Van Eeckhoutte, N. Bouckaert, A. Friart, J. Boreux, C. Arend, P. Evrard, L. Stefan, E. Hoffer, J. Herzet, M. Massoz, C. Celentano, M. Sprynger, L. Pierard, P. Melon, B. Van Hauwaert, C. Kuppens, D. Faes, D. Van Lier, A. Van Dorpe, A. Gerardy, O. Deceuninck, O. Xhaet, F. Dormal, E. Ballant, D. Blommaert, D. Yakova, M. Hristov, T. Yncheva, N. Stancheva, S. Tisheva, M. Tokmakova, F. Nikolov, D. Gencheva, T. Shalganov, B. Kunev, M. Stoyanov, D. Marchov, V. Gelev, V. Traykov, A. Kisheva, H. Tsvyatkov, R. Shtereva, S. Bakalska-Georgieva, S. Slavcheva, Y. Yotov, M. Kubíčková, A. Marni Joensen, A. Gammelmark, L. Hvilsted Rasmussen, P. Dinesen, S. Riahi, S. Krogh Venø, B. Sorensen, A. Korsgaard, K. Andersen, C. Fragtrup Hellum, A. Svenningsen, O. Nyvad, P. Wiggers, O. May, A. Aarup, B. Graversen, L. Jensen, M. Andersen, M. Svejgaard, S. Vester, S. Hansen, V. Lynggaard, M. Ciudad, R. Vettus, P. Muda, A. Maestre, S. Castaño, S. Cheggour, J. Poulard, V. Mouquet, S. Leparrée, J. Bouet, J. Taieb, A. Doucy, H. Duquenne, A. Furber, J. Dupuis, J. Rautureau, M. Font, P. Damiano, M. Lacrimini, J. Abalea, S. Boismal, T. Menez, J. Mansourati, G. Range, H. Gorka, C. Laure, C. Vassalière, N. Elbaz, N. Lellouche, K. Djouadi, F. Roubille, D. Dietz, J. Davy, M. Granier, P. Winum, C. Leperchois-Jacquey, H. Kassim, E. Marijon, J. Le Heuzey, J. Fedida, C. Maupain, C. Himbert, E. Gandjbakhch, F. Hidden-Lucet, G. Duthoit, N. Badenco, T. Chastre, X. Waintraub, M. Oudihat, J. Lacoste, C. Stephan, H. Bader, N. Delarche, L. Giry, D. Arnaud, C. Lopez, F. Boury, I. Brunello, M. Lefèvre, R. Mingam, M. Haissaguerre, M. Le Bidan, D. Pavin, V. Le Moal, C. Leclercq, O. Piot, T. Beitar, I. Martel, A. Schmid, N. Sadki, C. Romeyer-Bouchard, A. Da Costa, I. Arnault, M. Boyer, C. Piat, L. Fauchier, N. Lozance, S. Nastevska, A. Doneva, B. Fortomaroska Milevska, B. Sheshoski, K. Petroska, N. Taneska, N. Bakrecheski, K. Lazarovska, S. Jovevska, V. Ristovski, A. Antovski, E. Lazarova, I. Kotlar, J. Taleski, L. Poposka, S. Kedev, N. Zlatanovik, S. Jordanova, T. Bajraktarova Proseva, S. Doncovska, D. Maisuradze, A. Esakia, E. Sagirashvili, K. Lartsuliani, N. Natelashvili, N. Gumberidze, R. Gvenetadze, K. Etsadashvili, N. Gotonelia, N. Kuridze, G. Papiashvili, I. Menabde, S. Glöggler, A. Napp, C. Lebherz, H. Romero, K. Schmitz, M. Berger, M. Zink, S. Köster, J. Sachse, E. Vonderhagen, G. Soiron, K. Mischke, R. Reith, M. Schneider, W. Rieker, D. Boscher, A. Taschareck, A. Beer, D. Oster, O. Ritter, J. Adamczewski, S. Walter, A. Frommhold, E. Luckner, J. Richter, M. Schellner, S. Landgraf, S. Bartholome, R. Naumann, J. Schoeler, D. Westermeier, F. William, K. Wilhelm, M. Maerkl, R. Oekinghaus, M. Denart, M. Kriete, U. Tebbe, T. Scheibner, M. Gruber, A. Gerlach, C. Beckendorf, L. Anneken, M. Arnold, S. Lengerer, Z. Bal, C. Uecker, H. Förtsch, S. Fechner, V. Mages, E. Martens, H. Methe, T. Schmidt, B. Schaeffer, B. Hoffmann, J. Moser, K. Heitmann, S. Willems, S. Willems, C. Klaus, I. Lange, M. Durak, E. Esen, F. Mibach, H. Mibach, A. Utech, M. Gabelmann, R. Stumm, V. Ländle, C. Gartner, C. Goerg, N. Kaul, S. Messer, D. Burkhardt, C. Sander, R. Orthen, S. Kaes, A. Baumer, F. Dodos, A. Barth, G. Schaeffer, J. Gaertner, J. Winkler, A. Fahrig, J. Aring, I. Wenzel, S. Steiner, A. Kliesch, E. Kratz, K. Winter, P. Schneider, A. Haag, I. Mutscher, R. Bosch, J. Taggeselle, S. Meixner, A. Schnabel, A. Shamalla, H. Hötz, A. Korinth, C. Rheinert, G. Mehltretter, B. Schön, N. Schön, A. Starflinger, E. Englmann, G. Baytok, T. Laschinger, G. Ritscher, A. Gerth, D. Dechering, L. Eckardt, M. Kuhlmann, N. Proskynitopoulos, J. Brunn, K. Foth, C. Axthelm, H. Hohensee, K. Eberhard, S. Turbanisch, N. Hassler, A. Koestler, G. Stenzel, D. Kschiwan, M. Schwefer, S. Neiner, S. Hettwer, M. Haeussler-Schuchardt, R. Degenhardt, S. Sennhenn, S. Steiner, M. Brendel, A. Stoehr, W. Widjaja, S. Loehndorf, A. Logemann, J. Hoskamp, J. Grundt, M. Block, R. Ulrych, A. Reithmeier, V. Panagopoulos, C. Martignani, D. Bernucci, E. Fantecchi, I. Diemberger, M. Ziacchi, M. Biffi, P. Cimaglia, J. Frisoni, G. Boriani, I. Giannini, S. Boni, S. Fumagalli, S. Pupo, A. Di Chiara, P. Mirone, E. Fantecchi, G. Boriani, F. Pesce, C. Zoccali, V. L. Malavasi, A. Mussagaliyeva, B. Ahyt, Z. Salihova, K. Koshum-Bayeva, A. Kerimkulova, A. Bairamukova, E. Mirrakhimov, B. Lurina, R. Zuzans, S. Jegere, I. Mintale, K. Kupics, K. Jubele, A. Erglis, O. Kalejs, K. Vanhear, M. Burg, M. Cachia, E. Abela, S. Warwicker, T. Tabone, R. Xuereb, D. Asanovic, D. Drakalovic, M. Vukmirovic, N. Pavlovic, L. Music, N. Bulatovic, A. Boskovic, H. Uiterwaal, N. Bijsterveld, J. De Groot, J. Neefs, N. van den Berg, F. Piersma, A. Wilde, V. Hagens, J. Van Es, J. Van Opstal, B. Van Rennes, H. Verheij, W. Breukers, G. Tjeerdsma, R. Nijmeijer, D. Wegink, R. Binnema, S. Said, Ö. Erküner, S. Philippens, W. van Doorn, H. Crijns, T. Szili-Torok, R. Bhagwandien, P. Janse, A. Muskens, M. van Eck, R. Gevers, N. van der Ven, A. Duygun, B. Rahel, J. Meeder, A. Vold, C. Holst Hansen, I. Engset, D. Atar, B. Dyduch-Fejklowicz, E. Koba, M. Cichocka, A. Sokal, A. Kubicius, E. Pruchniewicz, A. Kowalik-Sztylc, W. Czapla, I. Mróz, M. Kozlowski, T. Pawlowski, M. Tendera, A. Winiarska-Filipek, A. Fidyk, A. Slowikowski, M. Haberka, M. Lachor-Broda, M. Biedron, Z. Gasior, M. Kołodziej, M. Janion, I. Gorczyca-Michta, B. Wozakowska-Kaplon, M. Stasiak, P. Jakubowski, T. Ciurus, J. Drozdz, M. Simiera, P. Zajac, T. Wcislo, P. Zycinski, J. Kasprzak, A. Olejnik, E. Harc-Dyl, J. Miarka, M. Pasieka, M. Ziemińska-Łuć, W. Bujak, A. Śliwiński, A. Grech, J. Morka, K. Petrykowska, M. Prasał, G. Hordyński, P. Feusette, P. Lipski, A. Wester, W. Streb, J. Romanek, P. Woźniak, M. Chlebuś, P. Szafarz, W. Stanik, M. Zakrzewski, J. Kaźmierczak, A. Przybylska, E. Skorek, H. Błaszczyk, M. Stępień, S. Szabowski, W. Krysiak, M. Szymańska, J. Karasiński, J. Blicharz, M. Skura, K. Hałas, L. Michalczyk, Z. Orski, K. Krzyżanowski, A. Skrobowski, L. Zieliński, M. Tomaszewska-Kiecana, M. Dłużniewski, M. Kiliszek, M. Peller, M. Budnik, P. Balsam, G. Opolski, A. Tymińska, K. Ozierański, A. Wancerz, A. Borowiec, E. Majos, R. Dabrowski, H. Szwed, A. Musialik-Lydka, A. Leopold-Jadczyk, E. Jedrzejczyk-Patej, M. Koziel, R. Lenarczyk, M. Mazurek, Z. Kalarus, K. Krzemien-Wolska, P. Starosta, E. Nowalany-Kozielska, A. Orzechowska, M. Szpot, M. Staszel, S. Almeida, H. Pereira, L. Brandão Alves, R. Miranda, L. Ribeiro, F. Costa, F. Morgado, P. Carmo, P. Galvao Santos, R. Bernardo, P. Adragão, G. Ferreira da Silva, M. Peres, M. Alves, M. Leal, A. Cordeiro, P. Magalhães, P. Fontes, S. Leão, A. Delgado, A. Costa, B. Marmelo, B. Rodrigues, D. Moreira, J. Santos, L. Santos, A. Terchet, D. Darabantiu, S. Mercea, V. Turcin Halka, A. Pop Moldovan, A. Gabor, B. Doka, G. Catanescu, H. Rus, L. Oboroceanu, E. Bobescu, R. Popescu, A. Dan, A. Buzea, I. Daha, G. Dan, I. Neuhoff, M. Baluta, R. Ploesteanu, N. Dumitrache, M. Vintila, A. Daraban, C. Japie, E. Badila, H. Tewelde, M. Hostiuc, S. Frunza, E. Tintea, D. Bartos, A. Ciobanu, I. Popescu, N. Toma, C. Gherghinescu, D. Cretu, N. Patrascu, C. Stoicescu, C. Udroiu, G. Bicescu, V. Vintila, D. Vinereanu, M. Cinteza, R. Rimbas, M. Grecu, A. Cozma, F. Boros, M. Ille, O. Tica, R. Tor, A. Corina, A. Jeewooth, B. Maria, C. Georgiana, C. Natalia, D. Alin, D. Dinu-Andrei, M. Livia, R. Daniela, R. Larisa, S. Umaar, T. Tamara, M. Loachim Popescu, D. Nistor, I. Sus, O. Coborosanu, N. Alina-Ramona, R. Dan, L. Petrescu, G. Ionescu, I. Popescu, C. Vacarescu, E. Goanta, M. Mangea, A. Ionac, C. Mornos, D. Cozma, S. Pescariu, E. Solodovnicova, I. Soldatova, J. Shutova, L. Tjuleneva, T. Zubova, V. Uskov, D. Obukhov, G. Rusanova, I. Soldatova, N. Isakova, S. Odinsova, T. Arhipova, E. Kazakevich, E. Serdechnaya, O. Zavyalova, T. Novikova, I. Riabaia, S. Zhigalov, E. Drozdova, I. Luchkina, Y. Monogarova, D. Hegya, L. Rodionova, L. Rodionova, V. Nevzorova, I. Soldatova, O. Lusanova, A. Arandjelovic, D. Toncev, M. Milanov, N. Sekularac, M. Zdravkovic, S. Hinic, S. Dimkovic, T. Acimovic, J. Saric, M. Polovina, T. Potpara, B. Vujisic-Tesic, M. Nedeljkovic, M. Zlatar, M. Asanin, V. Vasic, Z. Popovic, D. Djikic, M. Sipic, V. Peric, B. Dejanovic, N. Milosevic, A. Stevanovic, A. Andric, B. Pencic, M. Pavlovic-Kleut, V. Celic, M. Pavlovic, M. Petrovic, M. Vuleta, N. Petrovic, S. Simovic, Z. Savovic, S. Milanov, G. Davidovic, V. Iric-Cupic, D. Simonovic, M. Stojanovic, S. Stojanovic, V. Mitic, V. Ilic, D. Petrovic, M. Deljanin Ilic, S. Ilic, V. Stoickov, S. Markovic, S. Kovacevic, A. García Fernandez, A. Perez Cabeza, M. Anguita, L. Tercedor Sanchez, E. Mau, J. Loayssa, M. Ayarra, M. Carpintero, I. Roldán Rabadan, M. Leal, M. Gil Ortega, A. Tello Montoliu, E. Orenes Piñero, S. Manzano Fernández, F. Marín, A. Romero Aniorte, A. Veliz Martínez, M. Quintana Giner, G. Ballesteros, M. Palacio, O. Alcalde, I. García-Bolao, V. Bertomeu Gonzalez, F. Otero-Raviña, J. García Seara, J. Gonzalez Juanatey, N. Dayal, P. Maziarski, P. Gentil-Baron, D. Shah, M. Koç, E. Onrat, I. E. Dural, K. Yilmaz, B. Özin, S. Tan Kurklu, Y. Atmaca, U. Canpolat, L. Tokgozoglu, A. K. Dolu, B. Demirtas, D. Sahin, O. Ozcan Celebi, E. Diker, G. Gagirci, U. O. Turk, H. Ari, N. Polat, N. Toprak, M. Sucu, O. Akin Serdar, A. Taha Alper, A. Kepez, Y. Yuksel, A. Uzunselvi, S. Yuksel, M. Sahin, O. Kayapinar, T. Ozcan, H. Kaya, M. B. Yilmaz, M. Kutlu, M. Demir, C. Gibbs, S. Kaminskiene, M. Bryce, A. Skinner, G. Belcher, J. Hunt, L. Stancombe, B. Holbrook, C. Peters, S. Tettersell, A. Shantsila, D. Lane, K. Senoo, M. Proietti, K. Russell, P. Domingos, S. Hussain, J. Partridge, R. Haynes, S. Bahadur, R. Brown, S. McMahon, G. Lip, J. McDonald, K. Balachandran, R. Singh, S. Garg, H. Desai, K. Davies, W. Goddard, G. Galasko, I. Rahman, Y. Chua, O. Payne, S. Preston, O. Brennan, L. Pedley, C. Whiteside, C. Dickinson, J. Brown, K. Jones, L. Benham, R. Brady, L. Buchanan, A. Ashton, H. Crowther, H. Fairlamb, S. Thornthwaite, C. Relph, A. McSkeane, U. Poultney, N. Kelsall, P. Rice, T. Wilson, M. Wrigley, R. Kaba, T. Patel, E. Young, J. Law, C. Runnett, H. Thomas, H. McKie, J. Fuller, S. Pick, A. Sharp, A. Hunt, K. Thorpe, C. Hardman, E. Cusack, L. Adams, M. Hough, S. Keenan, A. Bowring, J. Watts, J. Zaman, K. Goffin, H. Nutt, Y. Beerachee, J. Featherstone, C. Mills, J. Pearson, L. Stephenson, S. Grant, A. Wilson, C. Hawksworth, I. Alam, M. Robinson, S. Ryan, R. Egdell, E. Gibson, M. Holland, D. Leonard, B. Mishra, S. Ahmad, H. Randall, J. Hill, L. Reid, M. George, S. McKinley, L. Brockway, W. Milligan, J. Sobolewska, J. Muir, L. Tuckis, L. Winstanley, P. Jacob, S. Kaye, L. Morby, A. Jan, T. Sewell, C. Boos, B. Wadams, C. Cope, P. Jefferey, N. Andrews, A. Getty, A. Suttling, C. Turner, K. Hudson, R. Austin, S. Howe, R. Iqbal, N. Gandhi, K. Brophy, P. Mirza, E. Willard, S. Collins, N. Ndlovu, E. Subkovas, V. Karthikeyan, L. Waggett, A. Wood, A. Bolger, J. Stockport, L. Evans, E. Harman, J. Starling, L. Williams, V. Saul, M. Sinha, L. Bell, S. Tudgay, S. Kemp, J. Brown, L. Frost, T. Ingram, A. Loughlin, C. Adams, M. Adams, F. Hurford, C. Owen, C. Miller, D. Donaldson, H. Tivenan, H. Button, A. Nasser, O. Jhagra, B. Stidolph, C. Brown, C. Livingstone, M. Duffy, P. Madgwick, P. Roberts, E. Greenwood, L. Fletcher, M. Beveridge, S. Earles, D. McKenzie, D. Beacock, M. Dayer, M. Seddon, D. Greenwell, F. Luxton, F. Venn, H. Mills, J. Rewbury, K. James, K. Roberts, L. Tonks, D. Felmeden, W. Taggu, A. Summerhayes, D. Hughes, J. Sutton, L. Felmeden, M. Khan, E. Walker, L. Norris, L. O’Donohoe, A. Mozid, H. Dymond, H. Lloyd-Jones, G. Saunders, D. Simmons, D. Coles, D. Cotterill, S. Beech, S. Kidd, B. Wrigley, S. Petkar, A. Smallwood, R. Jones, E. Radford, S. Milgate, S. Metherell, V. Cottam, C. Buckley, A. Broadley, D. Wood, J. Allison, K. Rennie, L. Balian, L. Howard, L. Pippard, S. Board, T. Pitt-Kerby

**Affiliations:** 1 Liverpool Centre for Cardiovascular Science, University of Liverpool and Liverpool Heart & Chest Hospital, Liverpool, UK; 2 Geriatric Unit, IRCCS Istituti Clinici Scientifici Maugeri, Milan, Italy; 3 Department of Clinical Sciences and Community Health, University of Milan, Milan, Italy; 4 Cardiology Division, Department of Biomedical, Metabolic and Neural Sciences, University of Modena and Reggio Emilia, Policlinico di Modena, Modena, Italy; 5 Clinical and Experimental Medicine PhD Program, University of Modena and Reggio Emilia, Modena, Italy; 6 Aalborg Thrombosis Research Unit, Department of Clinical Medicine, Aalborg University, Aalborg, Denmark; 7 Service de Cardiologie, Centre Hospitalier Universitaire Trousseau, Tours, France; 8 Department of Cardiology, Hospital Universitario Virgen de la Arrixaca, IMIB-Arrixaca, University of Murcia, CIBER-CV, Murcia, Spain; 9 Department of Cardiology, Ludwig-Maximilians-University, Munich, Germany; 10 School of Medicine, University of Belgrade, Belgrade, Serbia; 11 Intensive Arrhythmia Care, Cardiology Clinic, Clinical Center of Serbia, Belgrade, Serbia; 12 University of Medicine, ‘Carol Davila’, Colentina University Hospital, Bucharest, Romania

**Keywords:** Atrial fibrillation, Clinical phenotypes, Cluster analysis, Clinical management, Major adverse outcomes

## Abstract

**Background:**

Epidemiological studies in atrial fibrillation (AF) illustrate that clinical complexity increase the risk of major adverse outcomes. We aimed to describe European AF patients’ clinical phenotypes and analyse the differential clinical course.

**Methods:**

We performed a hierarchical cluster analysis based on Ward’s Method and Squared Euclidean Distance using 22 clinical binary variables, identifying the optimal number of clusters. We investigated differences in clinical management, use of healthcare resources and outcomes in a cohort of European AF patients from a Europe-wide observational registry.

**Results:**

A total of 9363 were available for this analysis. We identified three clusters: Cluster 1 (*n* = 3634; 38.8%) characterized by older patients and prevalent non-cardiac comorbidities; Cluster 2 (*n* = 2774; 29.6%) characterized by younger patients with low prevalence of comorbidities; Cluster 3 (*n* = 2955;31.6%) characterized by patients’ prevalent cardiovascular risk factors/comorbidities. Over a mean follow-up of 22.5 months, Cluster 3 had the highest rate of cardiovascular events, all-cause death, and the composite outcome (combining the previous two) compared to Cluster 1 and Cluster 2 (all *P* < .001). An adjusted Cox regression showed that compared to Cluster 2, Cluster 3 (hazard ratio (HR) 2.87, 95% confidence interval (CI) 2.27–3.62; HR 3.42, 95%CI 2.72–4.31; HR 2.79, 95%CI 2.32–3.35), and Cluster 1 (HR 1.88, 95%CI 1.48–2.38; HR 2.50, 95%CI 1.98–3.15; HR 2.09, 95%CI 1.74–2.51) reported a higher risk for the three outcomes respectively.

**Conclusions:**

In European AF patients, three main clusters were identified, differentiated by differential presence of comorbidities. Both non-cardiac and cardiac comorbidities clusters were found to be associated with an increased risk of major adverse outcomes.

**Supplementary Information:**

The online version contains supplementary material available at 10.1186/s12916-021-02120-3.

## Background

Atrial fibrillation (AF) is a cardiovascular condition which has a multifactorial origin, with several cardiovascular (CV) and non-CV risk factors and comorbidities significantly contributing to the development of incident AF cases [1–3]. Indeed, epidemiological data clearly demonstrates that the concomitant presence of multiple risk factors/comorbidities increases the risk of developing AF [2, 4]. Moreover, patients with AF have a high prevalence of various CV (i.e. heart failure, stroke, coronary artery disease, peripheral artery disease) and non-CV comorbidities, intended as non-cardiac or vascular related (i.e. diabetes mellitus, chronic kidney disease, gastric diseases, chronic obstructive pulmonary disease), as well as a high rate of multi-morbidity [4–7].

Cluster analysis is a data-driven approach which helps to improve clinical phenotype identification and classification, which has been applied to the study of several CV diseases [8–11]. Cluster analysis helps to identify the relevant clinical phenotypes, but has been applied to AF in relatively few studies [10–13]. In those studies which investigated this particular approach, cluster analysis helped to identify patients with similar clinical characteristics which were different between the various groups (more or less prevalence of risk factors and comorbidities combined together, i.e. ‘clinical phenotypes’), entailing differential management approach and differential risk for adverse outcomes, hence demonstrating how in groups of patients with different clinical characteristics AF can have a different clinical course [10–13].

Thus far, no large European AF cohort has been investigated to elucidate which are the most common clinical phenotypes in patients presenting with AF. Indeed, as demonstrated by previous literature, most of the cohorts examined so far were from North America and Asia [10–13]. Also a systematic review of machine-learning-based studies, an even more sophisticated form of cluster analysis, on disease definition and risk prediction found that most of the studies were based on North America cohorts [14].

The European Society of Cardiology (ESC) - European Heart Rhythm Association (EHRA) EURObservational Research Programme in AF (EORP-AF) General Long-Term Registry is the largest contemporary observational non-industry sponsored study on European AF patients presenting to cardiology centers. The aim of this report from the EORP-AF is to study the most relevant clinical phenotypes in terms of multi-morbidity clusters among European AF patients. Second, we aimed to analyse their impact in terms of clinical management, healthcare resources use, and major adverse outcomes.

## Methods

The ESC-EHRA EORP Atrial Fibrillation General Long-Term Registry is a multicenter observational registry held by the ESC and endorsed by the EHRA. The General Long-Term Registry has been preceded by the General Pilot Registry [15–18]. The EORP-AF General Long-Term Registry is a prospective, observational, multicenter registry established by the ESC in 27 participating countries. The study enrolled consecutive AF patients presenting in 250 cardiology practices, both in- and outpatient settings. The detailed description of the study design, baseline characteristics and 1-year follow-up results have been provided previously [19, 20]. Briefly, all AF patients enrolled had AF documented within 12 months before enrollment, based on electrocardiographic proof. All patients were ≥ 18 years old and provided written informed consent. Enrollment was undertaken from October 2013 to September 2016, with 1-year follow-up performed until to September 2018. Baseline and follow-up data were completed into a centralized electronic case report (eCRF) form by each investigator. According to its observational nature, only a limited set of variables, related to baseline thromboembolic and bleeding risk, baseline comorbidities and pharmacological therapy, had to be compulsory filled. ‘Unknown’ value, when provided, was considered as missing and then not considered. As reported in this study, more than 80% of patients reported a valid data for the core variables (Table 1 and Table 2). Patient data were obtained after the signing of a written informed consent by each patient, following the approval of study protocol by an Institutional Review Board/Ethic Committee. The study was firstly approved by the National Coordinators’ main institutions (listed in the Additional file 1) and subsequently was authorized by each peripheral site under the responsibility of the lead contact and study team (all listed in the Additional file 1), according to the specific national and local regulation. Any details regarding approval numbers for the study protocol regarding any specific site could be obtained from the Corresponding Authors, upon reasonable request. The study was performed according to the European Union Note for Guidance on Good Clinical Practice CPMP/ECH/135/95 and the Declaration of Helsinki.

**Table 1:** Baseline characteristics according to patient clusters

***N*** = 9363	Cluster 1 Non-CV comorbidities ***N*** = 3634	Cluster 2 Low risk ***N*** = 2774	Cluster 3 CV RFs/comorbidities ***N*** = 2955	***P***
**Age**, *years* Median [IQR]	73 [65–78]	65 [56–72]	73 [66–78]	< .001
**Age classes**, *n* (%)				< .001
*< 65 years*	865 (23.8)	1351 (48.7)	659 (22.3)	
*65–74 years*	1216 (33.5)	910 (32.8)	1039 (35.2)	
*75–84 years*	1282 (35.3)	441 (15.9)	1068 (36.1)	
*≥ 85 years*	271 (7.5)	72 (2.6)	189 (6.4)	
**Females**, *n* (%)	1604 (44.1)	964 (34.8)	1138 (38.5)	< .001
**Overweight/obese**, *n* (%)	755 (20.8)	1666 (60.1)	1619 (54.8)	< .001
**Smoking habit**, *n* (%)	1218 (33.5)	996 (35.9)	947 (32.0)	0.008
**Alcohol habit**, *n* (%)	1088 (33.7)	1004 (41.0)	786 (29.8)	< 0.001
**Reason for admission**, *n* (%)				< .001
*AF*	2427 (66.8)	2299 (82.9)	1571 (53.2)	
*Other CV*	1034 (28.5)	400 (14.4)	1221 (41.3)	
*Other non-CV*	173 (4.8)	75 (2.7)	163 (5.5)	
**Site of admission**, *n* (%)				< .001
*Hospital facility*	1783 (49.1)	1261 (45.5)	1650 (55.8)	
*Outpatient facility*	1851 (50.9)	1513 (54.5)	1305 (44.2)	
**Type of AF**, *n* (%) *9360*				< .001
*First detected*	547 (15.1)	532 (19.2)	373 (12.6)	
*Paroxysmal*	936 (25.8)	865 (31.2)	692 (23.4)	
*Persistent*	693 (19.1)	641 (23.1)	496 (16.8)	
*Long-standing persistent*	143 (3.9)	114 (4.1)	134 (4.5)	
*Permanent*	1256 (34.6)	577 (20.8)	1210 (41.0)	
*Unknown*	58 (1.6)	44 (1.6)	49 (1.7)	
**CHA**_**2**_**DS**_**2**_**-VASc score**, median [IQR] *9358*	3 [2–4]	2 [1–3]	4 [3–5]	< .001
**CHA**_**2**_**DS**_**2**_**-VASc quartiles**, *n* (%)				< .001
*Q1 (0–2)*	1253 (34.5)	1834 (66.2)	520 (17.6)	
*Q2 (3)*	887 (24.4)	483 (17.4)	598 (20.3)	
*Q3 (4)*	777 (21.4)	312 (11.3)	734 (24.9)	
*Q4 (≥ 5)*	716 (19.7)	143 (5.2)	1101 (37.3)	
**High TE risk**, *n* (%) *9358*	2865 (78.9)	1351 (48.7)	2771 (93.8)	< .001
**HAS-BLED score**, median [IQR]	2 [1–2]	1 [0–2]	2 [1–3]	< .001
**High bleeding risk**, *n* (%)	616 (17.0)	172 (6.2)	809 (27.4)	< .001
**EHRA score**, median [IQR] *9362*	2 [1–2]	2 [1–2]	2 [1–2]	.002
**EHRA II-IV**, *n* (%) *9362*	1892 (52.1)	1613 (58.2)	1536 (52.0)	< .001
**Previous stroke**, n (%)	358 (9.9)	40 (1.4)	157 (5.3)	< .001
**Previous thromboembolic events**, *n* (%)	529 (14.6)	193 (7.0)	325 (11.0)	< .001
**Hypertension**, *n* (%)	2230 (61.4)	1253 (45.2)	2266 (76.7)	< .001
**Heart failure**, *n* (%)	1327 (36.5)	419 (15.1)	1658 (56.1)	< .001
**Diabetes mellitus**, *n* (%)	297 (8.2)	177 (6.4)	1613 (54.6)	< .001
**Lipid disorder**, *n* (%)	1356 (38.4)	874 (32.5)	1512 (52.5)	< .001
**Congenital heart disease**, *n* (%)	50 (1.4)	24 (0.9)	29 (1.0)	.24
**Valvular disease**, *n* (%)	2200 (60.5)	615 (22.2)	1724 (58.3)	< .001
**CAD**, *n* (%)	1093 (30.1)	273 (9.8)	1311 (44.4)	< .001
**CIED implant,** *n* (%)	364 (10.2)	166 (6.1)	400 (13.7)	< 0.001
**Chronic kidney disease**, *n* (%)	138 (3.8)	58 (2.1)	911 (30.8)	< .001
**History of bleeding**, *n* (%)	311 (8.6)	23 (0.8)	156 (5.3)	< .001
**COPD**, *n* (%)	446 (12.3)	80 (2.9)	267 (9.0)	< .001
**Anaemia**, *n* (%)	176 (4.8)	14 (0.5)	268 (9.1)	< .001
**Predisposition to bleeding**, *n* (%)	76 (2.1)	18 (0.6)	81 (2.7)	< .001
**Peripheral arterial disease**, *n* (%)	297 (8.2)	131 (4.7)	318 (10.8)	< .001
**Liver disease**, *n* (%)	125 (3.4)	17 (0.6)	79 (2.7)	< .001
**OSAS**, *n* (%)	83 (2.3)	230 (8.3)	127 (4.3)	< .001
**Neoplasm**, *n* (%)	513 (14.1)	40 (1.4)	162 (5.5)	< .001
**Hyperthyroidism**, *n* (%)	305 (8.4)	47 (1.7)	84 (2.8)	< .001
**Hypothyroidism**, *n* (%)	509 (14.0)	49 (1.8)	334 (11.3)	< .001
**Multi-morbidity**, *n* (%)	3151 (86.9)	1312 (47.3)	2877 (97.6)	< .001
**Comorbidities**, *N* Median [IQR]	3 [2–4]	1 [0–2]	4 [3–6]	
**Comorbidities quartiles**, *n* (%)				< .001
*Q1 (0–2)*	1289 (35.5)	2134 (76.9)	337 (11.4)	
*Q2 (3)*	901 (24.8)	313 (11.3)	518 (17.6)	
*Q3 (4–5)*	1024 (28.2)	272 (9.8)	1174 (39.8)	
*Q4 (≥ 6)*	414 (11.4)	55 (2.0)	918 (31.2)	
**Frailty**, *n* (%)	571 (16.5)	185 (7.0)	1003 (35.5)	< .001
**Polypharmacy**, *n* (%) *9301*	1801 (49.9)	941 (34.1)	2162 (73.7)	< .001
**Drugs**, *N* Median [IQR]	4 [3–6]	4 [2–5]	6 [4–7]	
**Drug quartiles**, *n* (%)				< .001
*Q1 (0–3)*	1053 (29.2)	1272 (46.2)	345 (11.8)	
*Q2 (4–5)*	1457 (40.3)	1018 (36.9)	1034 (35.2)	
*Q3 (6)*	564 (15.6)	264 (9.6)	589 (20.1)	
*Q4 (≥ 7)*	537 (14.9)	202 (7.3)	966 (32.9)	

Legend: *AF* atrial fibrillation, *CAD* coronary artery disease, *CIED* cardiac implantable electronic device, *CKD* chronic kidney disease, *COPD* chronic obstructive pulmonary disease, *CV* cardiovascular, *IQR* interquartile range, *OSAS* obstructive sleep apnoea syndrome, *PAD* peripheral artery disease, *RFs* risk factors, *TE* thromboembolic

**Table 2:** Atrial fibrillation clinical management according to patient clusters

	Cluster 1	Cluster 2	Cluster 3	***P***
***Antithrombotic treatment***				
**Any antiplatelet**, *n* (%) 9356	708 (19.5)	304 (11.0)	812 (27.5)	< .001
**Any OAC**, *n* (%) 9359	3143 (86.5)	2306 (83.2)	2563 (86.7)	< .001
**Any VKA**, *n* (%) 9358	1833 (50.5)	1163 (42.0)	1639 (55.5)	< .001
**Any NOAC**, *n* (%) 9354	1314 (36.2)	1145 (41.3)	925 (31.3)	< .001
**Antithrombotic pattern**, *n* (%)				< .001
*No antithrombotic*	217 (6.0)	318 (11.5)	142 (4.8)	
*Only antiplatelet*	273 (7.5)	147 (5.3)	250 (8.5)	
*Only VKA*	1524 (42.0)	1059 (38.2)	1228 (41.6)	
*Only NOAC*	1182 (32.6)	1089 (39.3)	773 (26.2)	
*Antiplatelet + OAC*	435 (12.0)	157 (5.7)	562 (19.0)	
***Primary management (before admission)***				< .001
**Primary management strategy**, *n* (%) *7753*				< .001
*Rate control*	1371 (45.3)	802 (36.5)	1317 (52.0)	
*Rhythm control*	1203 (39.8)	1083 (49.3)	852 (33.7)	
*Observation*	452 (14.9)	311 (14.2)	362 (14.3)	
**Primary ECV**, *n* (%) *7350*	693 (24.1)	604 (28.5)	476 (20.2)	< .001
**Primary PCV**, *n* (%) *7281*	730 (25.6)	629 (29.9)	621 (26.6)	.003
**Primary catheter ablation**, *n* (%) *7531*	171 (5.8)	181 (8.4)	108 (4.4)	< .001
***Management during admission/consultation***				<.001
**Intervention planned/performed**, *n* (%) *9363*	1285 (34.6)	1150 (41.5)	984 (33.3)	<.001
**ECV**, *n* (%) *3392*	566 (45.0)	628 (54.6)	379 (38.5)	< .001
**PCV**, *n* (%) *3392*	304 (24.2)	204 (17.7)	245 (24.9)	< .001
**Catheter ablation**, *n* (%) *3392*	176 (14.0)	261 (22.7)	100 (10.2)	< .001
**Management strategy at discharge**, *n* (%)				< .001
*Rate control*	1724 (47.5)	984 (35.5)	1522 (51.7)	
*Rhythm control*	1365 (37.6)	1333 (48.1)	981 (33.3)	
*Observation*	537 (14.8)	452 (16.3)	442 (15.0)	
**ABC pathway adherence**, *n* (%)	666 (30.8)	655 (34.5)	431 (26.2)	< .001

Legend: *ECV* electrical cardioversion, *NOAC* non-vitamin K antagonist oral anticoagulant, *OAC* oral anticoagulant, *PCV* pharmacological cardioversion, *VKA* vitamin K antagonist

Symptomatic status was defined according to EHRA score [1]. Thromboembolic risk was defined according to CHA_2_DS_2_-VASc score [1]. Bleeding risk was defined according to HAS-BLED score [1]. Both CHA_2_DS_2_-VASc and HAS-BLED scores were computed according to the original schemes. High thromboembolic risk was defined as CHA_2_DS_2_-VASc ≥ 2 in males and ≥ 3 in females. High bleeding risk was defined for HAS-BLED ≥ 3. We have also examined distribution of CHA_2_DS_2_-VASc quartiles across the clusters. Multi-morbidity was defined as the concomitant presence of at least 2 different comorbidities [21]. Frailty was defined based on a 40-item frailty index ≥ 0.25 built according to Rockwood and Mitnitski [22]. Polypharmacy was defined as the concomitant use of ≥ 5 drugs [23]. Additionally, we examined the distribution of comorbidities and concomitant drug distribution. Adherence to the Atrial fibrillation Better Care (ABC) pathway was defined according to a previously published study [24]. Briefly, the ABC pathway has been proposed to streamline integrated care and holistic management in AF patients and is based on the following: (i) avoid stroke with anticoagulation; (ii) better symptom management with patient-centred symptom-directed decisions on rate or rhythm control; (iii) cardiovascular risk factor and comorbidity optimization including lifestyle changes [25]. Adherence to the ABC pathway has consistently been associated with reduction in risk for major clinical outcomes associated with AF [26]. According to the eCRF rate/rhythm control strategy, as well the use of specific medical techniques (electric or pharmacological cardioversion, catheter ablation), were evaluated both priori admission/consultation and during admission/consultation. All the baseline variables were established regardless of the clustering process and according to previous international definitions; hence, no a priori difference can be determined according to the various clusters.

### Clustering process

We performed an agglomerative hierarchical cluster analysis based on Ward’s Minimum Variance Method to minimize the total within-cluster variance and we selected the squared Euclidean as measure of distance or dissimilarity. The squared Euclidean distance was used since only dichotomous variables were selected. The aim of the analysis was to identify the optimal number of clusters that were homogenous and indicative of a clinically relevant phenotypic subgroup of AF patients without a priori knowledge of the outcomes. We a priori selected 22 clinical variables as follows: age, sex, heart failure, coronary artery disease, valvular disease, hypertension, diabetes mellitus, ischemic stroke, peripheral ischemic events, liver disease, chronic obstructive pulmonary disease, anaemia, dementia, any cardiomyopathy, hyperthyroidism, hypothyroidism, chronic kidney disease, obstructive sleep apnoea syndrome, malignancy, body mass index. All variables were considered as categorical—either present or absent. Age and body mass index were dichotomized, according to usual clinical practice, as age < 75 and ≥ 75 years and body mass index < 25 kg/m^2^ (normal BMI) and ≥ 25 kg/m^2^ (overweight/obese).

From the EORP-AF dataset, a total of 9363 (84.8%) were found to have all data available for the 22 variables and were included in the analysis. The clustering algorithm begins with each element (i.e. patient) in a separate cluster and then proceeds with a ‘bottom-up’ approach grouping each cluster with the most similar one until all clusters become one. The hierarchical clustering process is visually represented by a dendrogram graph in which vertical lines represent clusters that are joined together and the position of an horizontal line on the scale indicates the rescaled distance at which clusters were joined (the higher is the rescaled distance at which clusters combine on the *y*-axis, the more dissimilarity exists between clusters since they joined nearer to the final point of the dendrogram, in which the clusters become one). Ward Linkage coefficients provided a mean to determine the heterogeneity between clusters by providing the difference in Euclidean distances over which clusters are joined (i.e. the difference between subsequent horizontal lines on the dendrogram) with larger distances indicating greater heterogeneity between the clusters joined at that step. By examining the dendrogram produced by the clustering process and considering the Ward Linkage coefficients, we found that the distance between the points in which the elements grouped together (between 10 and 15 on the *y*-axis) became larger and consequently the groupings became more heterogeneous after being expanded to 3 clusters. Therefore, the 3-cluster model was used in this study.

### Outcomes

To evaluate the comprehensive impact of different AF clinical phenotypes, we considered a large set of outcomes. First, we examined the differential use of healthcare resources according to the three clusters. In patients enrolled during hospitalization, we evaluated the overall length of stay. Further, we recorded and analysed the occurrence of cardiology and internal medicine/general practitioner visits, as well as emergency room admissions (all intended as a binary variable as ‘at least one visit/admission’), in the three clusters identified, occurred separately during the first and second year of follow-up. Second, we considered several major clinical adverse events, throughout the follow-up period. The primary clinical outcomes were as follows: (i) cardiovascular events including the occurrence of any thromboembolic event (including stroke, transient ischemic attack and any peripheral embolism), any acute coronary syndrome and CV death; (ii) all-cause death; and (iii) a final composite outcome of CV events and/or all-cause death. All primary outcomes were analysed with a time-to-event and intention-to-treat approach, with observation censored after the first event occurred. Additionally, we evaluated the occurrence of several secondary clinical outcomes: (i) any bleeding; and hospital readmission for (ii) any cause; (iii) CV-related; (iv) AF; (v) cardiovascular but non-AF related; and (vi) any non-CV cause. These secondary outcomes, given the lack of dates, were not analysed with a time-to-event approach. All outcomes were assessed by clinical visit or telephonic interview with patient or next of kind and reported by investigators. Each event was not centrally adjudicated but categorized according to investigator’s clinical evaluation. All data regarding outcomes were collected before the analysis was planned and performed; hence, no difference in assessment of outcomes exists according to the clusters.

### Statistical analysis

Continuous variables were expressed as mean and standard deviation or median and IQR, and differences across the clusters were evaluated according to one-way analysis of variance (ANOVA) and Kruskal-Wallis one-way ANOVA, respectively. Categorical variables were expressed as counts and percentages and differences across clusters were evaluated according to the chi-squared test. A logistic regression model, adjusted for type of AF and EHRA score, was compiled to examine the association between clusters and use of oral anticoagulant (OAC) therapy.

To evaluate the differences in length of hospital stay between the three clusters, a one-way analysis of co-variance (ANCOVA) model, adjusted for type of AF and EHRA score, was used. To analyze the association between clusters and other healthcare use resources, a logistic regression model was used, adjusted for type of AF, EHRA score and use of OAC.

Differences in cumulative risk for the three main study outcomes were evaluated using log-rank test and drafted according to Kaplan-Meier curves. To investigate the association between the three clusters and the study primary clinical outcomes, a Cox regression analysis, adjusted for type of AF, EHRA score and use of OAC. The association between the three clusters and the secondary clinical outcomes utilized a logistic regression model, adjusted for type of AF, EHRA score and use of OAC.

Finally, to understand whether the application of a more comprehensive and integrated clinical management could have had an impact on the occurrence of clinical outcomes, we performed an analysis to assess the impact of adherence to the ABC pathway on the composite outcome of CV events and all-cause death, according to the three clusters. A Cox regression model for ABC vs. non-ABC and each ABC criterion, adjusted for type of AF, EHRA score and use of OAC, was performed. All logistic regression analysis results were reported as odds ratio (OR) and 95% confidence interval (CI). All Cox regression analysis results were reported as hazard ratio (HR) and 95% CI. No formal interaction analysis was performed, and missing data were just considered as missing with no imputation analysis performed. A two-sided *p* < 0.05 was considered statistically significant. All analyses were performed using SPSS statistical software version 25.0.0.1 (IBM, NY, USA) for MacOS.

## Results

Among the overall 11,096 patients originally enrolled in the study, a total of 9363 (84.8%) were included in this analysis. The 1733 remaining patients were excluded based on missing data regarding the variables used for the clustering process, Median [IQR] age was 71 [62-77], with 3706 (39.6%) females and a median [IQR] CHA_2_DS_2_-VASc equal to 3 [2–4] (Table 1). According to the methods described above, we identified 3 clusters, characterized as follows.

### Cluster 1—older patients with non-cardiac comorbidities

A total of 3634 (38.8%) patients were in Cluster 1. Median [IQR] age was 73 [65-78] years, with 1553 (42.7%) patients being ≥ 75 years and the largest proportion of females compared to the other clusters (*P* < .001). Conversely, the proportion of overweight/obese patients was the lowest in Cluster 1 compared to Clusters 2 and 3 (*P* < .001). Patients in Cluster 1 had a clinical history more burdened with previous stroke, thromboembolic and bleeding events (*P* < .001) and a higher prevalence of several non-cardiovascular comorbidities, compared to other clusters (Table 1). Mean CHA_2_DS_2_-VASc and HAS-BLED scores were higher than in patients in Cluster 2, but lower than in patients in Cluster 3 (both *P* < .001). In terms of CHA_2_DS_2_-VASc score quartiles, the proportion of patients in Quartile 1 (Q1) (i.e. CHA_2_DS_2_-VASc 0-2) was highest in Cluster 2, while was lowest in Cluster 3 (Table 1).

### Cluster 2—younger patients with few comorbidities

A total of 2774 (29.6%) patients were grouped in Cluster 2. This cluster included younger patients (median [IQR] age 65 [56-72] years) compared to the other two clusters (*P* < .001). This cluster had the largest proportion of overweight/obese patients, who were also more likely to be admitted for AF as primary diagnosis and enrolled at an outpatient facility. Moreover, patients in this cluster were those more likely smoking and drinking alcohol. In addition, patients in Cluster 2 were more likely to have first detected and paroxysmal AF and were more symptomatic, but less likely to have permanent AF (*P* < .001) than those in the other clusters. Patients in Cluster 2 generally had less comorbidities than patients in other clusters (*P* < .001). Thromboembolic and bleeding risks were the lowest, together with prevalence of multi-morbidity, polypharmacy and frailty among patients in Cluster 2.

### Cluster 3—older patients with high prevalence of cardiovascular risk factors and comorbidities

A total of 2955 (31.6%) patients were included in Cluster 3. Median [IQR] age was 73 [66-78] years, with 1257 (42.5%) females. Patients in Cluster 3 were more likely to be admitted for cardiovascular reasons other than AF (*P* < .001) and more commonly had permanent AF compared to other clusters (*P* < .001). Cluster 3 patients were more likely to be enrolled at hospital admission (*P* < .001). Patients in Cluster 3 had more prevalent CV risk factors and clinical history of cardiac and vascular disease, with 2771 (93.8%) patients with high thromboembolic risk and 809 (27.4%) with high bleeding risk. In terms of CHA_2_DS_2_-VASc quartiles, patients in Q4 (i.e. CHA_2_DS_2_-VASc ≥ 5) were more prevalent in Cluster 3, and least so in Cluster 2 (Table 1). Accordingly, these patients reported the highest prevalence of previous cardiac implantable electronic device. Additionally, the prevalence of multi-morbidity, frailty and polypharmacy was highest in Cluster 3 compared to the other clusters (all *P* < .001). When examining the median number of comorbidities and concomitant drugs, both were highest in Cluster 3 and progressively lower in Cluster 1 followed by Cluster 2. When looking at quartiles of comorbidities and concomitant drugs, patients in Q4 (respectively comorbidities ≥ 6 and concomitant drugs ≥ 7) were more commonly found in Cluster 3, and lower in Clusters 1 and 2 (Table 1).

**Fig. 1:**
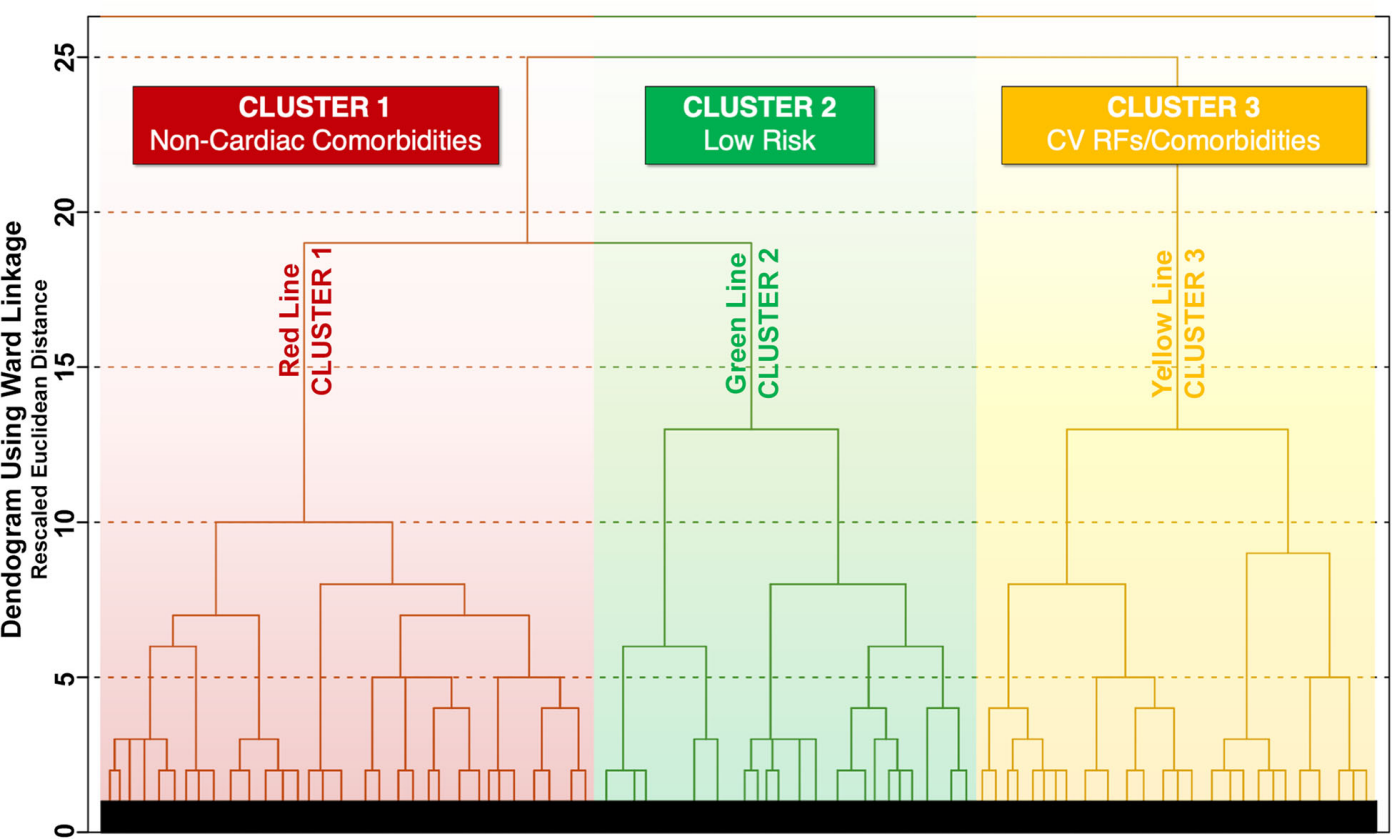
Patients’ cluster membership. Legend: CV = cardiovascular; RFs = risk factors

Based on baseline characteristics, we can ‘label’ the three clusters (Fig. 1) as follows: (i) Cluster 1: older patients with prevalent non-cardiac comorbidities; (ii) Cluster 2: younger patients with an overall low thromboembolic risk and low comorbidity burden; and (iii) Cluster 3: patients with prevalent cardiovascular risk factors and comorbidities, at highest risk of adverse events.

### Management of AF

Analysis of the management of AF according to the three clusters is reported in Table 2. Use of antiplatelet drugs was highest in Cluster 3 (*P* < .001), while use of OAC was lowest in Cluster 2 (*P* < .001). Among OAC, vitamin K antagonists were more likely used in Cluster 3, with non-vitamin K antagonist OACs use more prevalent in Cluster 2 (both *P* < .001). Dual antithrombotic therapy was more used in Cluster 3 (*P* < .001). When considering only those patients eligible for OAC treatment (male patients with CHA_2_DS_2_-VASc ≥ 1 or female patients with CHA_2_DS_2_-VASc ≥ 2), we found substantially similar prevalence of treatments, with the only exception of prevalence of OAC which was higher in Cluster 2 than in the others (*P* = .004) (Additional file 1, Table S1).

After adjustment for type of AF and EHRA score, compared to those in Cluster 2, both patients in Cluster 1 and in Cluster 3 were more likely prescribed with OAC (OR 1.20, 95% CI 1.05–1.39 and OR 1.17, 95% CI 1.01–1.36, respectively). Among OAC users, Cluster 1 and Cluster 3 were significantly associated with greater vitamin K antagonist use compared to non-vitamin K antagonist OACs, when compared to Cluster 2 (adjusted OR 1.21, 95% CI 1.08–1.36 and OR 1.45, 95% CI 1.29–1.63, respectively).

Prior admission/consultation, a rate control strategy occurred more often among patients in Cluster 3, while a rhythm control strategy was more often used in Cluster 2 (*P* < .001). All rhythm control strategies, except for pharmacological cardioversion, were more prevalent in Cluster 2 than in the other clusters (*P* < .001, *P* = .003 and *P* < .001, respectively).

During the index admission/consultation, a rhythm control intervention was planned/performed more frequently in Cluster 2 (*P* < .001). Electrical cardioversion and catheter ablation were more likely used in Cluster 2, while pharmacological cardioversion was more common in Clusters 1 and 3 (all P < .001). At discharge, a rate control strategy was more likely used in Cluster 3 and patients in Cluster 3 were less likely managed as adherent to ABC pathway (*P* < .001).

### Use of healthcare resources

Among the 4694 patients enrolled during a hospital admission, mean [standard deviation] length of stay was progressively lower in patients in Cluster 3 (8.07 [8.50] days), Cluster 1 (6.52 [7.29] days) and Cluster 2 (4.36 [6.33] days) (*P* < .001 for the overall model and for differences between each cluster). After adjustment for EHRA score and type of AF, differences in overall length of stay remained significant (*F* = 72.215, *P* < .001).

During follow-up, use of healthcare resources (Table 3) differed significantly among the three clusters. Patients in Cluster 1 and Cluster 3 were more likely to have at least one internal medicine/general practitioner visit both at 1 year and 2 years, even after adjustments (see Table 3). Patients in Cluster 3 were more likely to have at least one emergency room visit within the first 12 months of follow-up (OR 1.50, 95% CI 1.29–1.75).

**Table 3:** Health resource use during follow-up according to patient clusters

	Cluster 1	Cluster 2	Cluster 3	***P***
**Cardiology visits 1Y**, *n* (%)	2227 (75.1)	1802 (74.5)	1693 (73.3)	.34
OR [95% CI]*	1.06 [0.93–1.20]	Ref.	0.97 [0.85–1.20]	–
**Internal medicine/GP visits 1Y**, *n* (%)	1208 (51.3)	909 (46.5)	1006 (52.4)	< .001
OR [95% CI]*	**1.24 [1.10–1.40]**	Ref.	**1.30 [1.14–1.48]**	–
**ER admissions 1Y**, *n* (%)	494 (17.1)	390 (16.7)	490 (21.9)	< .001
OR [95% CI]*	1.08 [0.93–1.26]	Ref.	**1.50 [1.29–1.75]**	–
**Cardiology visits 2Y**, *n* (%)	1788 (68.9)	1442 (68.2)	1355 (68.6)	.88
OR [95% CI]*	1.03 [0.91–1.17]	Ref.	1.00 [0.88–1.15]	–
**Internal medicine/GP visits 2Y**, *n* (%)	1124 (51.0)	808 (45.5)	876 (51.2)	.001
OR [95% CI]*	**1.27 [1.18–1.44]**	Ref.	**1.29 [1.13–1.48]**	–
**ER admissions 2Y**, *n* (%)	352 (14.1)	287 (14.0)	297 (15.7)	.25
OR [95% CI]*	1.02 [0.86–1.21]	Ref.	1.16 [0.97–1.39]	–

*Legend:* *adjusted for type of AF, EHRA score, use of OAC; *1Y* 1 year follow-up, *2Y* 2 years follow-up, *CI* confidence interval, *ER* emergency room, *GP* general practitioner, *OR* odds ratio. For other acronyms, please see previous tables’ legends

### Major adverse events

**Fig. 2:**
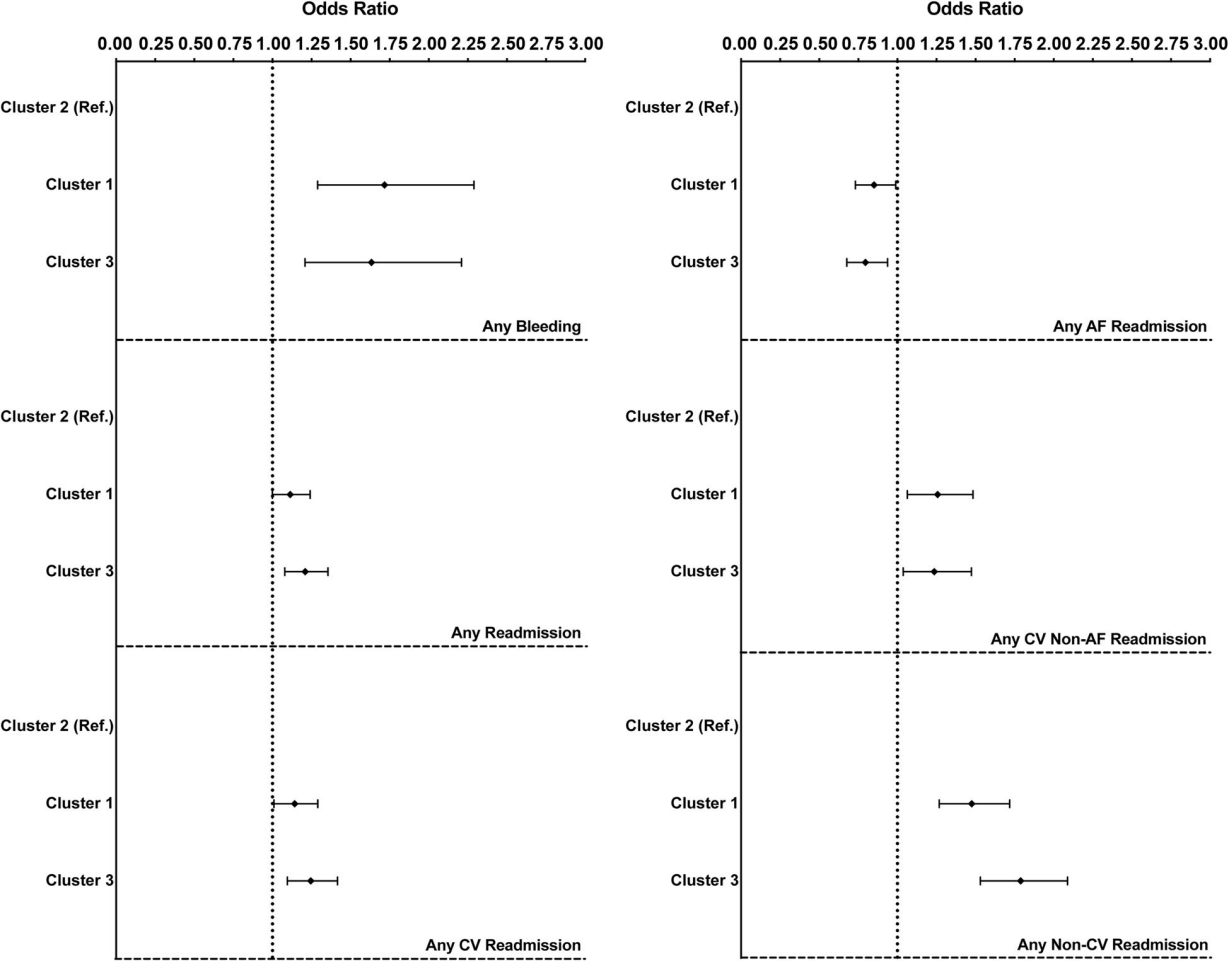
Multivariable logistic regression analysis for secondary clinical outcomes. Legend: adjusted for type of AF, EHRA score, use of OAC; AF = atrial fibrillation; CV = cardiovascular; OAC = oral anticoagulant

Outcomes were assessed over a median [IQR] 731 [701-749] days of follow-up (Table 4). A progressively higher rate of events was found from Cluster 2 to Cluster 1 and Cluster 3 for the occurrence of cardiovascular events, all-cause death, composite outcomes and any cardiovascular non-AF-related hospital readmission (all *P* < .001) (Table 4). Occurrence of any bleeding and any non-CV-related hospital readmission was significantly lower in Cluster 2, while a higher rate of AF-related readmission was found. A non-significant trend for a higher rate of any readmission and any cardiovascular readmission was evident for Cluster 3 (Table 4). Adjusted logistic regression analyses (Fig. 2) found a higher risk for all the secondary outcomes in Cluster 1 and Cluster 3, except the risk for any AF-related readmission, which was lower for both these clusters.

**Table 4:** Major adverse clinical events according to patient clusters

***N*** = 8701	Cluster 1	Cluster 2	Cluster 3	***P***
**Cardiovascular events**, *n* (%)	295 (8.7)	121 (4.7)	385 (14.1)	< .001
**All-cause death**, *n* (%)	331 (9.8)	101 (3.9)	370 (13.6)	< .001
**Composite outcome**, *n* (%)	502 (14.9)	187 (7.2)	571 (20.9)	< .001
**Any bleeding**, *n* (%)	156 (4.6)	71 (2.7)	121 (4.5)	< .001
**Any readmission**, *n* (%)	1264 (37.7)	940 (36.3)	1063 (39.3)	.09
**Any CV readmission**, *n* (%)	816 (24.4)	592 (22.9)	694 (25.6)	.06
**Any AF readmission**, *n* (%)	413 (12.3)	396 (15.3)	304 (11.2)	< .001
**Any CV non-AF readmission**, *n* (%)	546 (16.3)	306 (11.8)	516 (19.1)	< .001
**Any non-CV readmission**, *n* (%)	406 (12.1)	260 (10.0)	322 (11.9)	.03

*Legend:* for acronyms, please see previous tables’ legends

**Fig. 3:**
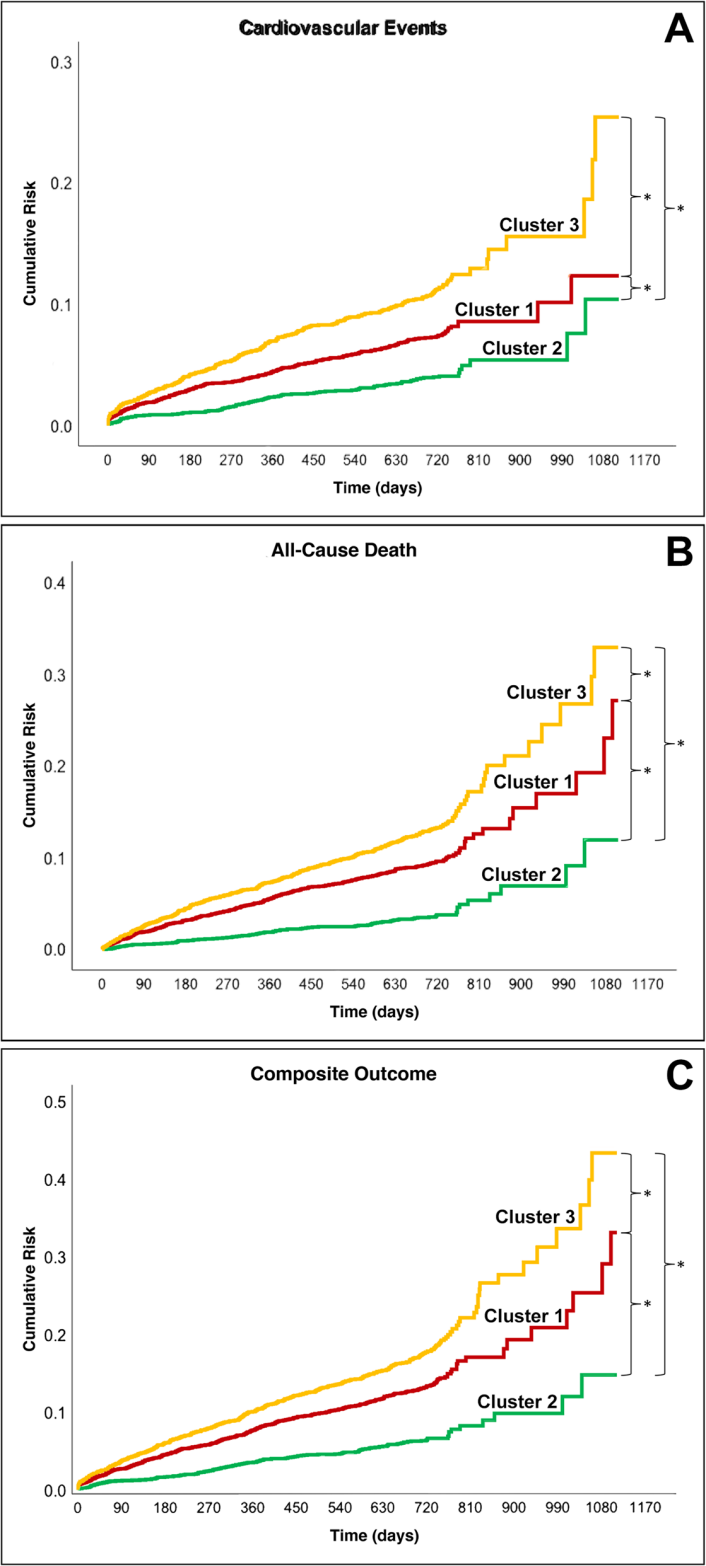
Kaplan-Meier curves for primary clinical study outcomes. Legend: **A** Cardiovascular events = log-rank 85.975, *P* < .001. **B** All-cause death = log-rank 132.790, *P* < .001. **C** Composite outcome = log-rank 132.997, *P* < .001. All pairwise comparisons were significant for *P* < .001. Green line = Cluster 2; orange line = Cluster 1; yellow line = Cluster 3

Regarding the main clinical study outcomes, Kaplan-Meier curves (Fig. 3) show a progressively higher cumulative risk across the three clusters for all the main study outcomes. Adjusted Cox regression analyses (Table 5) found that compared to Cluster 1, Cluster 2 and Cluster 3 were associated with a progressively higher risk for all the three study primary outcomes.

**Table 5:** Cox regression analysis for main study outcomes

	Univariate		Multivariable^**a**^	
	HR (95% CI)	***P***	HR (95% CI)	***P***
**Cardiovascular Events,** ***n*** **(%)**				
Cluster 1	1.85 [1.46–2.34]	< .001	1.88 [1.48–2.38]	< .001
*Cluster 2 (Ref.)*	–	–	–	–
Cluster 3	2.82 [2.24–3.55]	< .001	2.87 [2.27–3.62]	< .001
**All-cause death,** ***n*** **(%)**				
Cluster 1	2.55 [2.03–3.21]	< .001	2.50 [1.98–3.15]	< .001
*Cluster 2 (Ref.)*	–	–	–	–
Cluster 3	3.55 [2.83–4.46]	< .001	3.42 [2.72–4.31]	< .001
**Composite outcome,** ***n*** **(%)**				
Cluster 1	2.09 [1.74–2.51]	< .001	2.09 [1.74–2.51]	< .001
*Cluster 2 (Ref.)*	–	–	–	–
Cluster 3	2.81 [2.34–3.37]	< .001	2.79 [2.32–3.35]	< .001

*Legend:* ^a^adjusted for type of AF, EHRA score, use of OAC. *HR* hazard ratio. For other acronyms, please see previous tables’ legends

### Adherence to ABC pathway and outcomes according to clusters (Table 6)

In Cluster 1, we found that while the adherence to the overall ABC pathway was not significantly associated with a lower risk of the composite outcome, the ‘B’ criterion showed a non-significant trend in inverse association with the risk of event occurrence. In Cluster 2, which was at a generally low thromboembolic risk, adherence to ABC pathway was found to be associated to a lower risk for the composite outcome, with no single criterion being independently associated with lower risk. Cluster 3 showed that full adherence to the ABC pathway was strongly associated with a significant reduction in the risk of adverse outcomes, but that the risk reduction was mainly associated with adherence to the ‘C’ criterion (Table 6).

**Table 6:** Adherence to ABC pathway and outcomes according to clusters

	Composite outcome		
	HR^a^	95% CI	*P*
***Cluster 1***			
ABC vs. Non-ABC	0.89	0.66–1.21	.45
A Criterion	1.25	0.80–1.96	.34
B Criterion	0.59	0.34–1.01	.06
C Criterion	0.82	0.63–1.05	.12
***Cluster 2***			
ABC vs. Non-ABC	0.62	0.40–0.97	.04
A Criterion	1.23	0.78–1.95	.38
B Criterion	0.63	0.28–1.41	.26
C Criterion	0.55	0.37–0.81	.002
***Cluster 3***			
ABC vs. Non-ABC	0.53	0.36–0.76	< .001
A Criterion	1.14	0.38–3.38	.82
B Criterion	0.96	0.57–1.62	.87
C Criterion	0.71	0.54–0.93	.01

*Legend:* ^a^adjusted for type of AF, EHRA score, use of OAC. For acronyms, please see previous tables’ legends

## Discussion

In this cluster analysis derived from the ESC-EHRA EORP-AF General Long-Term Registry, we showed that three main clinical phenotypes can be identified among European AF patients. The first cluster was characterized by older patients with a prevalent high burden of non-cardiac comorbidities (Cluster 1); the second cluster was associated with a younger age, with a low burden of comorbidities and an overall low thromboembolic risk (Cluster 2); in the third cluster, we observed older AF patients with a high burden of CV risk factors and comorbidities, with an overall high burden of multi-morbidity and frailty, and the highest thromboembolic risk (Cluster 3). The three clusters showed clear differences in terms of OAC therapy and clinical management, with a differential risk in long-term major adverse events. Both Cluster 1 and Cluster 3 showed an overall higher use of healthcare resources during follow-up and a higher risk of major adverse events, particularly those patients in Cluster 3.

Recently, machine-learning-based data analysis has been increasing applied to biomedical scientific research, even in cardiovascular health [27]. Among the more basic machine-learning analyses, the unsupervised cluster analysis has garnered attention, with studies in the hypertension and heart failure cohorts [8, 28]. Use of this analytic technique allows us to perform insightful epidemiological analysis, allowing better risk stratification, which could lead to more focused management and treatment [8]. Thus far, cluster analysis in AF patients has been applied to AF patients only in two large observational studies, the ‘Outcomes Registry for Better Informed Treatment of Atrial Fibrillation’ and the ‘Keio Interhospital Cardiovascular Studies for AF’ registry; US and Japanese cohorts, respectively [10, 11]. More recently other two cluster analyses regarding large AF datasets were published [12, 13]. In this context, our study provides novel evidence, representing the first large cluster analysis focused on European AF patients.

The current analyses demonstrate how the level and the type of comorbidities are key essential elements in differentiating AF patients with distinctive clinical needs and long-term risks. Previous studies investigating cluster analysis in AF patients have shown how specific clusters characterized by a higher burden of comorbidities and risk factors are associated with higher risk of major adverse events during long-term follow-up [10–13]. The results we provide not only underline the importance of the role of comorbidities in determining the occurrence of major adverse events, but also highlight the differential impact of non-CV and CV comorbidities. While on one side some previous analyses regarding the use of machine-learning systems, of which cluster analysis represents a primordial representative, underlined how several methodological issues can limit the reliability and reproducibility of such data, it is our opinion that putting our data in the context of previous literature helps to stress some important concepts about how not only comorbidities are crucial in determining the risk of outcomes, but is also important how they associate each other and influence the natural history of the disease as a whole.

The Framingham Heart Study previously showed that AF patients with comorbidities have a consistently increased risk for cardiovascular events and all-cause death compared to those without [29]. In an analysis from the ‘Apixaban for Reduction in Stroke and Other Thromboembolic Events in Atrial Fibrillation’ trial, multi-morbidity was found to be associated with an increased risk of adverse outcomes [7]. Furthermore, in a registry-based analysis of hospitalized AF patients, an increasing Charlson Comorbidity Index, a validated tool to evaluate the level of multi-morbidity, was directly associated with the occurrence of stroke, major bleeding and all-cause death [6]. Our cluster analysis demonstrates that not all the comorbidities carry the same risk. Indeed, while Cluster 1 demonstrates an increased risk, Cluster 3 showed a significantly greater risk compared to Cluster 1.

The 2020 ESC guidelines on the management of AF [1] introduce a paradigm shift promoting a more integrated and holistic approach to AF diagnosis, characterization and management, summarized as ‘CC to ABC’. If the first ‘C’ relates to confirmation of AF diagnosis, the second ‘C’ focused specifically on the need to properly evaluate and characterize each AF patient, in order to appropriately stratify their individual risk and plan the best diagnostic and therapeutic pathway. Regarding the ‘characterization’ of AF patients, the ESC guidelines recommends the ‘4S-AF scheme’ to provide a ‘structured characterization of AF and to streamline the assessment of AF patients at different healthcare levels, inform treatment decision-making and facilitate optimal management of AF patients’ [30]. The 4S-AF scheme suggests evaluating patients as follows: (i) stroke risk; (ii) symptom severity; (iii) severity of AF burden; (iv) substrate severity. Fully supporting the 4S-AF approach, our analysis demonstrates how comprehensive clinical characterization provides important information, delineating a clear profile for each cluster, which also differentiated patients in terms of healthcare needs and long-term risks. Hence, the information gathered through the clustering process can help in defining the patients’ risks and determine strategies to improve their care and management.

The differential impact of the ABC pathway underlines how the same treatment strategies can have distinct effects according to the clinical profile of the patient. Also, the evidence that the ABC pathway is more effective in the cluster with the greatest multi-morbidity (Cluster 3), and that the effectiveness is driven largely by the management of comorbidities, emphasizes the prominent role of CV risk factors/comorbidities in determining adverse outcomes in AF patients, while clearly demonstrating how a comprehensive management plan is clearly needed to improve patient care, as well as a proper evaluation and characterization.

Our data are also in line with more recent research in the area of multi-morbidity, which now distinguishes patterns/clusters of conditions, clearly defined in terms of sociodemographic, clinical and functional characteristics, beyond the mere presence of multiple conditions [31]. This analysis represents the first application of this approach to a large European AF population.

Results from the cluster analysis in AF patients underline the importance of stratifying patients’ characteristics and identify those clinical phenotypes more prone to adverse events, beyond the mere focus on thromboembolic risk, and to properly address patients’ care need and healthcare management plan. If the clustering process is not easily applicable in clinical daily life, information gathered from this analysis reinforces the concept that the presence of comorbidities increases the risk. Thus, we further reinforce the need for implementation of the 4S-AF scheme to characterize AF, which allows an easy and straightforward way for everyday clinical use, also helping to the use and evaluation of quality indicators [32]. We believe it is important to underline that our data are generated from a large Europe-wide cohort. Even though patients were gathered principally from third-level cardiology practices, they were collected consecutively over the enrolment time and with a minimal set of inclusion/exclusion criteria, reassuring on the representativity of our data. Even though we based our clustering analysis on a lower number of variables, we think that the superimposable results, in particular about the main characteristics of the clusters and their impact on risk of outcomes, could reassure about the reliability of our analysis, with a relevant generalisability of the results provided.

### Limitations

The main limitation of the study relates to its observational nature, with a limited power to detect differences in subgroups which were not pre-specified in the study design. Moreover, as an observational registry, completeness of data is not as high as clinical trial; hence, this aspect may have partially limited our analytical capability. Notwithstanding, several paper already published about the EORP-AF General Long-Term Registry showed similar findings to other contemporary registries held in other geographical locations, both in terms of baseline characteristics and follow-up data [19, 20]. Second, the data presented do not imply causality, rather they describe a statistical association. Furthermore, identified clusters may vary according to patients’ characteristics and available data and may change over time, since risk is dynamic (changing with ageing and incident risk factors [33–35]) and not a ‘one-off’ assessment. Moreover, no formal interaction analysis was performed, and missing data were just considered as missing with no imputation analysis performed. Finally, the optimal number of clusters can be difficult to determine since different statistical algorithms may generate different results and the final selection of clusters was based in part on investigator discretion, also no analysis regarding the between-cluster heterogeneity could have been performed. The extent of the limitations suggests caution in interpreting our findings. Notwithstanding, we believe that given the large cohort presented generated from the entire European territory confers a significant generalizability, even though is necessary to take in mind the possible limitations of such analyses [14].

## Conclusions

In European AF patients, three main clinical clusters were identified, older patients with non-cardiac comorbidities, a younger, low risk group and older patients with cardiac comorbidities. Both non-cardiac and cardiac comorbidities clusters were found to be associated with an increased risk of major adverse outcomes.

## Supplementary Information

**Additional file 1 : Table S1**. Use of Antithrombotic drugs according to Patient Clusters in patients with High Thromboembolic Risk. STROBE Checklist. **Appendix.** EURObservational Research Programme Atrial Fibrillation (EORP-AF) Long-Term General Registry Committees and Investigators.

## Data Availability

All relevant data regarding the study are included in the manuscript.

## Abbreviations

ABC: Atrial fibrillation Better Care
AF: Atrial fibrillation
CI: Confidence interval
CV: Cardiovascular
EHRA: European Heart Rhythm Association
EORP: EURObservational Research Programme
ESC: European Society of Cardiology
HR: Hazard ratio
OAC: Oral anticoagulant
OR: Odds ratio
