# Supplementary material for: Impact of clinical phenotypes on management and outcomes in European atrial fibrillation patients: a report from the ESC-EHRA EURObservational Research Programme in AF (EORP-AF) General Long-Term Registry
Source: BMC Med. 2021 Oct 20;19:256. doi: 10.1186/s12916-021-02120-3 (PMC8527730; doi:10.1186/s12916-021-02120-3)
Supplement: Supplementary file 1 — Additional file 1 : Table S1. Use of Antithrombotic drugs according to Patient Clusters in patients with High Thromboembolic Risk. STROBE Checklist. Appendix. EURObservational Research Programme Atrial Fibrillation (EORP-AF) Long-Term General Registry Committees and Investigators. [file 12916_2021_2120_MOESM1_ESM.docx]

**Impact of Clinical Phenotypes on Management and Outcomes in European Atrial Fibrillation Patients: A report from the ESC-EHRA EURObservational Research Programme in AF (EORP-AF) General Long-Term Registry**

**Running Title:** Cluster Analysis in AF

Marco Proietti MD PhD, Marco Vitolo MD, Stephanie L Harrison PhD,

Deirdre A Lane PhD, Laurent Fauchier MD PhD, Francisco Marin MD PhD,

Michael Nabauer MD, Tatjana S Potpara MD PhD, Gheorghe-Andrei Dan MD, Giuseppe Boriani MD PhD, Gregory Y H Lip MD

on behalf of the ESC-EHRA EORP-AF Long-Term General Registry Investigators

*Additional File 1*

**Table S1: Use of Antithrombotic drugs according to Patient Clusters in patients with High Thromboembolic Risk**

|  | **Cluster 1** | **Cluster 2** | **Cluster 3** | **P** |
| --- | --- | --- | --- | --- |
| **Any Antiplatelet**, n (%) 6984 | 670 (19.7) | 239 (11.3) | 809 (27.5) | <.001 |
| **Any OAC**, n (%) 6985 | 2983 (87.8) | 189 (89.9) | 2552 (86.8) | .004 |
| **Any VKA**, n (%) 6985 | 1749 (51.5) | 994 (47.1) | 1635 (55.6) | <.001 |
| **Any NOAC**, n (%) 6982 | 1238 (36.5) | 905 (42.9) | 918 (31.2) | <.001 |
| **Antithrombotic Pattern**, n (%) 6984  *No Antithrombotic*  *Only Antiplatelet*  *Only VKA*  *Only NOAC*  *Antiplatelet + OAC* | 167 (4.9)  247 (7.3)  1448 (42.7)  1110 (32.7)  423 (12.5) | 118 (5.6)  96 (4.5)  898 (42.5)  856 (40.5)  143 (6.8) | 140 (4.8)  249 (8.5)  1225 (41.7)  767 (26.1)  560 (19.0) | <.001 |

**Legend:** NOAC= Non-Vitamin K Antagonist Oral Anticoagulant; OAC= Oral Anticoagulant; VKA= Vitamin K Antagonist.

**STROBE Checklist**

|  | Item No | Recommendation | Page No |
| --- | --- | --- | --- |
| **Title and abstract** | 1 | (*a*) Indicate the study’s design with a commonly used term in the title or the abstract | 1 |
|  |  | (*b*) Provide in the abstract an informative and balanced summary of what was done and what was found | 2 |
| Introduction | | | |
| Background/rationale | 2 | Explain the scientific background and rationale for the investigation being reported | 4-5 |
| Objectives | 3 | State specific objectives, including any prespecified hypotheses | 5 |
| Methods | | | |
| Study design | 4 | Present key elements of study design early in the paper | 5-6 |
| Setting | 5 | Describe the setting, locations, and relevant dates, including periods of recruitment, exposure, follow-up, and data collection | 5-6 |
| Participants | 6 | (*a*) Give the eligibility criteria, and the sources and methods of selection of participants. Describe methods of follow-up | 5,6,9 |
|  |  | (*b*) For matched studies, give matching criteria and number of exposed and unexposed |  |
| Variables | 7 | Clearly define all outcomes, exposures, predictors, potential confounders, and effect modifiers. Give diagnostic criteria, if applicable | 9,10 |
| Data sources/ measurement | 8* | For each variable of interest, give sources of data and details of methods of assessment (measurement). Describe comparability of assessment methods if there is more than one group | 6,7,8 |
| Bias | 9 | Describe any efforts to address potential sources of bias | 10-11 |
| Study size | 10 | Explain how the study size was arrived at | 12 |
| Quantitative variables | 11 | Explain how quantitative variables were handled in the analyses. If applicable, describe which groupings were chosen and why | 6,7,8 |
| Statistical methods | 12 | (*a*) Describe all statistical methods, including those used to control for confounding | 10-11 |
|  |  | (*b*) Describe any methods used to examine subgroups and interactions |  |
|  |  | (*c*) Explain how missing data were addressed |  |
|  |  | (*d*) If applicable, explain how loss to follow-up was addressed |  |
|  |  | (*e*) Describe any sensitivity analyses |  |
| Results | | |  |
| Participants | 13* | (a) Report numbers of individuals at each stage of study—eg numbers potentially eligible, examined for eligibility, confirmed eligible, included in the study, completing follow-up, and analysed | 12 |
|  |  | (b) Give reasons for non-participation at each stage |  |
|  |  | (c) Consider use of a flow diagram |  |
| Descriptive data | 14* | (a) Give characteristics of study participants (eg demographic, clinical, social) and information on exposures and potential confounders | 12-14 |
|  |  | (b) Indicate number of participants with missing data for each variable of interest |  |
|  |  | (c) Summarise follow-up time (eg, average and total amount) |  |
| Outcome data | 15* | Report numbers of outcome events or summary measures over time | 15-17 |

| Main results | 16 | (*a*) Give unadjusted estimates and, if applicable, confounder-adjusted estimates and their precision (eg, 95% confidence interval). Make clear which confounders were adjusted for and why they were included | 15,16, 39, 41 |
| --- | --- | --- | --- |
|  |  | (*b*) Report category boundaries when continuous variables were categorized |  |
|  |  | (*c*) If relevant, consider translating estimates of relative risk into absolute risk for a meaningful time period |  |
| Other analyses | 17 | Report other analyses done—eg analyses of subgroups and interactions, and sensitivity analyses | 16,17,42 |
| Discussion | | | |
| Key results | 18 | Summarise key results with reference to study objectives | 17 |
| Limitations | 19 | Discuss limitations of the study, taking into account sources of potential bias or imprecision. Discuss both direction and magnitude of any potential bias | 21,22 |
| Interpretation | 20 | Give a cautious overall interpretation of results considering objectives, limitations, multiplicity of analyses, results from similar studies, and other relevant evidence | 21,22 |
| Generalisability | 21 | Discuss the generalisability (external validity) of the study results | 21,22 |
| Other information | | | |
| Funding | 22 | Give the source of funding and the role of the funders for the present study and, if applicable, for the original study on which the present article is based | 25 |

**APPENDIX**

**EURObservational Research Programme Atrial Fibrillation (EORP-AF) Long-Term General Registry Committees and Investigators**

**Executive committee:** G. Boriani (Chair), G.Y.H. Lip, L. Tavazzi, A. P. Maggioni, G-A. Dan, T. Potpara, M. Nabauer, F. Marin, Z. Kalarus, L. Fauchier, R. Ferrari, A. Shantsila.

**Steering Committee (National Coordinators):** A. Goda, *University Hospital Center "Mother Tereza", Tirana, Albania*; G. Mairesse, *Cliniques du Sud-Luxembourg, Arlon, Belgium;* T. Shalganov, *National Heart Hospital, Sofia, Bulgaria;* L. Antoniades, *Nicosia General Hospital, Latsia, Cyprus;* M. Taborsky, *University Hospital Olomouc, Olomouc, Czech Republic;* S. Riahi, *Aalborg University Hospital, Aalborg, Denmark;* P. Muda, *University of Tartu, Tartu, Estonia;* I. García Bolao, *Navarra Institute for Health Research, Pamplona, Spain;* O. Piot, *Centre Cardiologique du Nord, Saint-Denis, France;* M. Nabauer, *Ludwig-Maximilians-University, Munich, Germany;* K. Etsadashvili, *G. Chapidze Emergency Cardiology Center, Tbilisi, Georgia;* EN. Simantirakis, *University Hospital of Heraklion, School of Medicine, University of Crete, Heraklion, Crete, Greece;* M. Haim, *Soroka Medical Center, Beer Sheva, Israel;* A. Azhari, J. Najafian, *Cardiovascular Research Institute, Isfahan University of Medical Sciences, Isfahan, Iran;* M. Santini, *San Filippo Neri Hospital, Rome, Italy;* E. Mirrakhimov, *National Center of Cardiology and Internal Medicine, Bishkek, Kyrgyzstan;* K. Kulzida, *Scientific-Research Institute of Cardiology and Internal Diseases, Almaty, Republic of Kazakhstan;* A. Erglis, *Pauls Stradins Clinical University Hospital University of Latvia Riga Latvia;* L. Poposka, *University Clinic of Cardiology, Faculty of Medicine, Ss Cyril and Methodius University of Skopje, Skopje, Republic of Macedonia;* MR. Burg, *Mater Dei Hospital, Triq Dun Karm Psaila, Malta;* H. Crijns, Ö. Erküner, *Cardiovascular Research Institute Maastricht (CARIM), Maastricht University Medical Centre, Maastricht, The Netherlands;* D. Atar, *Oslo University Hospital Ullevål and Institute of Clinical Sciences, University of Oslo, Oslo, Norway;* R. Lenarczyk, *Silesian Center for Heart Disease, Zabrze, Poland;* M. Martins Oliveira, *Hospital Santa Marta, Lisbon, Portugal;* D. Shah, *Department of Medicine Specialities, University Hospital Geneva, Geneva, Switzerland;* G-A. Dan, *Colentina University Hospital, Bucharest, Romania;* E. Serdechnaya, *Northern State Medical University, Arkhangelsk, Russia;* T. Potpara, *Cardiology Clinic, Clinical Center of Serbia, Belgrade, Serbia;* E. Diker, *Başakşehir Çam and Sakura City Hospital, Istanbul, Turkey;* G.Y.H. Lip, D. Lane; *City Hospital, University of Birmingham, Birmingham, United Kingdom.*

**Investigators:** **ALBANIA** Durrës: E. Zëra, Tirana: U. Ekmekçiu, V. Paparisto, M. Tase, Tirana: H. Gjergo, J. Dragoti, A. Goda, **BELGIUM** Bastogne: M. Ciutea, N. Ahadi, Z. el Husseini, M. Raepers, Gilly: J. Leroy, P. Haushan, A. Jourdan, Haine Saint Paul: C. Lepiece, Hasselt: L. Desteghe, J. Vijgen, P. Koopman, G. Van Genechten, H. Heidbuchel, Kortrijk: T. Boussy, M. De Coninck, H. Van Eeckhoutte, N. Bouckaert, La Louviere: A. Friart, J. Boreux, C. Arend, Liege: P. Evrard, Liège: L. Stefan, E. Hoffer, J. Herzet, M. Massoz, Liège: C. Celentano, M. Sprynger, L. Pierard, Liège: P. Melon, Overpelt: B. Van Hauwaert, C. Kuppens, D. Faes, D. Van Lier, A. Van Dorpe, Waremme: A. Gerardy, Yvoir: O. Deceuninck, O. Xhaet, F. Dormal, E. Ballant, D. Blommaert, **BULGARIA** Pleven: D. Yakova, M. Hristov, T. Yncheva, N. Stancheva, S. Tisheva, Plovdiv: M. Tokmakova, F. Nikolov, D. Gencheva, Sofia: T. Shalganov, B. Kunev, M. Stoyanov, Sofia: D. Marchov, V. Gelev, V. Traykov, Varna: A. Kisheva, H. Tsvyatkov, R. Shtereva, S. Bakalska-Georgieva, S. Slavcheva, Y. Yotov, **CZECH REPUBLIC** Ústí nad Labem: M. Kubíčková, **DENMARK** Aalborg: A. Marni Joensen, A. Gammelmark, L. Hvilsted Rasmussen, P. Dinesen, S. Riahi, S. Krogh Venø, B. Sorensen, A. Korsgaard, K. Andersen, C. Fragtrup Hellum, Esbjerg: A. Svenningsen, O. Nyvad, P. Wiggers, Herning: O. May, A. Aarup, B. Graversen, L. Jensen, M. Andersen, M. Svejgaard, S. Vester, S. Hansen, V. Lynggaard, Madrid: M. Ciudad, Tallinn: R. Vettus, Tartu: P. Muda, **ESTONIA** Elche, Alicante: A. Maestre, Toledo: S. Castaño, **FRANCE** Abbeville: S. Cheggour, Abbeville: J. Poulard, V. Mouquet, S. Leparrée, Aix-en-Provence: J. Bouet, J. Taieb, Amiens: A. Doucy, H. Duquenne, Angers: A. Furber, J. Dupuis, J. Rautureau, Aurillac: M. Font, P. Damiano, Avignon Cedex: M. Lacrimini, Brest: J. Abalea, S. Boismal, T. Menez, J. Mansourati, Chartres: G. Range, H. Gorka, C. Laure, C. Vassalière, Creteil: N. Elbaz, N. Lellouche, K. Djouadi, Montpellier: F. Roubille, D. Dietz, J. Davy, Nimes: M. Granier, P. Winum, C. Leperchois-Jacquey, Paris: H. Kassim, E. Marijon, J. Le Heuzey, Paris: J. Fedida, C. Maupain, C. Himbert, E. Gandjbakhch, F. Hidden-Lucet, G. Duthoit, N. Badenco, T. Chastre, X. Waintraub, M. Oudihat, J. Lacoste, C. Stephan, Pau: H. Bader, N. Delarche, L. Giry, Pessac: D. Arnaud, C. Lopez, F. Boury, I. Brunello, M. Lefèvre, R. Mingam, M. Haissaguerre, Rennes: M. Le Bidan, D. Pavin, V. Le Moal, C. Leclercq, Saint Denis: O. Piot, T. Beitar, Saint Etienne: I. Martel, A. Schmid, N. Sadki, C. Romeyer-Bouchard, A. Da Costa, Tours: I. Arnault, M. Boyer, C. Piat, L. Fauchier, **FYR MACEDONIA** Bitola: N. Lozance, S. Nastevska, Ohrid: A. Doneva, B. Fortomaroska Milevska, B. Sheshoski, K. Petroska, N. Taneska, N. Bakrecheski, Skopje: K. Lazarovska, S. Jovevska, V. Ristovski, A. Antovski, Skopje: E. Lazarova, I. Kotlar, J. Taleski, L. Poposka, S. Kedev, Skopje: N. Zlatanovik, Štip: S. Jordanova, T. Bajraktarova Proseva, S. Doncovska, **GEORGIA** Tbilisi: D. Maisuradze, A. Esakia, E. Sagirashvili, K. Lartsuliani, N. Natelashvili, N. Gumberidze, R. Gvenetadze, Tbilisi: K. Etsadashvili, N. Gotonelia, N. Kuridze, Tbilisi: G. Papiashvili, I. Menabde, **GERMANY** Aachen: S. Glöggler, A. Napp, C. Lebherz, H. Romero, K. Schmitz, M. Berger, M. Zink, S. Köster, J. Sachse, E. Vonderhagen, G. Soiron, K. Mischke, Bad Reichenhall: R. Reith, M. Schneider, Berlin: W. Rieker, Biberach: D. Boscher, A. Taschareck, A. Beer, Boppard: D. Oster, Brandenburg: O. Ritter, J. Adamczewski, S. Walter, Chemnitz: A. Frommhold, E. Luckner, J. Richter, M. Schellner, S. Landgraf, S. Bartholome, Chemnitz: R. Naumann, J. Schoeler, Dachau: D. Westermeier, F. William, K. Wilhelm, M. Maerkl, Detmold: R. Oekinghaus, M. Denart, M. Kriete, U. Tebbe, Ebersbach: T. Scheibner, Erlangen: M. Gruber, A. Gerlach, C. Beckendorf, L. Anneken, M. Arnold, S. Lengerer, Z. Bal, C. Uecker, H. Förtsch, S. Fechner, V. Mages, Friedberg: E. Martens, H. Methe, Göttingen: T. Schmidt, Hamburg: B. Schaeffer, B. Hoffmann, J. Moser, K. Heitmann, S. Willems, S. Willems, Hartmannsdorf: C. Klaus, I. Lange, Heidelberg: M. Durak, E. Esen, Itzehoe: F. Mibach, H. Mibach, Kassel: A. Utech, Kirchzarten: M. Gabelmann, R. Stumm, V. Ländle, Koblenz: C. Gartner, C. Goerg, N. Kaul, S. Messer, D. Burkhardt, C. Sander, R. Orthen, S. Kaes, Köln: A. Baumer, F. Dodos, Königsbrück: A. Barth, G. Schaeffer, Leisnig: J. Gaertner, J. Winkler, Leverkusen: A. Fahrig, J. Aring, I. Wenzel, Limburg: S. Steiner, A. Kliesch, E. Kratz, K. Winter, P. Schneider, Ludwigsburg: A. Haag, I. Mutscher, R. Bosch, Markkleeberg: J. Taggeselle, S. Meixner, Meissen: A. Schnabel, Meppen: A. Shamalla, H. Hötz, A. Korinth, Merzig: C. Rheinert, Moosburg: G. Mehltretter, Mühldorf: B. Schön, N. Schön, A. Starflinger, E. Englmann, Munich: G. Baytok, T. Laschinger, G. Ritscher, Munich: A. Gerth, Münster: D. Dechering, L. Eckardt, Nienburg: M. Kuhlmann, N. Proskynitopoulos, Paderborn: J. Brunn, K. Foth, Pirna: C. Axthelm, H. Hohensee, K. Eberhard, S. Turbanisch, Plauen: N. Hassler, A. Koestler, Riesa: G. Stenzel, Riesa: D. Kschiwan, M. Schwefer, S. Neiner, S. Hettwer, Rotenburg a.d. Fulda: M. Haeussler-Schuchardt, R. Degenhardt, S. Sennhenn, S. Steiner, Starnberg: M. Brendel, Westerstede: A. Stoehr, W. Widjaja, S. Loehndorf, A. Logemann, J. Hoskamp, J. Grundt, Zorneding: M. Block, Zwiesel: R. Ulrych, A. Reithmeier, V. Panagopoulos, **ITALY** Bologna: C. Martignani, D. Bernucci, E. Fantecchi, I. Diemberger, M. Ziacchi, M. Biffi, P. Cimaglia, J. Frisoni, G. Boriani, Firenze: I. Giannini, S. Boni, S. Fumagalli, S. Pupo, A. Di Chiara, P. Mirone, Modena: E. Fantecchi, G. Boriani, F. Pesce, C. Zoccali, V.L. Malavasi, **KAZAKHSTAN** Almaty: A. Mussagaliyeva, B. Ahyt, Z. Salihova, K. Koshum-Bayeva, **KYRGYZSTAN** Bishkek: A. Kerimkulova, A. Bairamukova, E. Mirrakhimov, **LATVIA** Riga: B. Lurina, R. Zuzans, S. Jegere, I. Mintale, K. Kupics, K. Jubele, A. Erglis, O. Kalejs, **MALTA** Birkirkara: K. Vanhear, M. Burg, M. Cachia, E. Abela, S. Warwicker, T. Tabone, R. Xuereb, **MONTENEGRO** Podgorica: D. Asanovic, D. Drakalovic, M. Vukmirovic, N. Pavlovic, L. Music, N. Bulatovic, A. Boskovic, **NETHERLANDS** Almere: H. Uiterwaal, N. Bijsterveld, Amsterdam: J. De Groot, J. Neefs, N. van den Berg, F. Piersma, A. Wilde, Delfzijl: V. Hagens, Enschede: J. Van Es, J. Van Opstal, B. Van Rennes, H. Verheij, W. Breukers, Heerenveen: G. Tjeerdsma, R. Nijmeijer, D. Wegink, R. Binnema, Hengelo: S. Said, Maastricht: Ö. Erküner, S. Philippens, W. van Doorn, H. Crijns, Rotterdam: T. Szili-Torok, R. Bhagwandien, P. Janse, A. Muskens, s-Hertogenbosch: M. van Eck, R. Gevers, N. van der Ven, Venlo: A. Duygun, B. Rahel, J. Meeder, **NORWAY** Oslo: A. Vold, C. Holst Hansen, I. Engset, D. Atar, **POLAND** Bytom: B. Dyduch-Fejklowicz, E. Koba, M. Cichocka, Cieszyn: A. Sokal, A. Kubicius, E. Pruchniewicz, Gliwice: A. Kowalik-Sztylc, W. Czapla, Katowice: I. Mróz, M. Kozlowski, T. Pawlowski, M. Tendera, Katowice: A. Winiarska-Filipek, A. Fidyk, A. Slowikowski, M. Haberka, M. Lachor-Broda, M. Biedron, Z. Gasior, Kielce: M. Kołodziej, M. Janion, Kielce: I. Gorczyca-Michta, B. Wozakowska-Kaplon, Łódź: M. Stasiak, P. Jakubowski, T. Ciurus, J. Drozdz, Łódź: M. Simiera, P. Zajac, T. Wcislo, P. Zycinski, J. Kasprzak, Nysa: A. Olejnik, E. Harc-Dyl, J. Miarka, M. Pasieka, M. Ziemińska-Łuć, W. Bujak, Opoczno: A. Śliwiński, A. Grech, J. Morka, K. Petrykowska, M. Prasał, Opole: G. Hordyński, P. Feusette, P. Lipski, A. Wester, Radlin: W. Streb, Rzeszów: J. Romanek, P. Woźniak, M. Chlebuś, P. Szafarz, W. Stanik, Szczecin: M. Zakrzewski, J. Kaźmierczak, Szczecin: A. Przybylska, E. Skorek, H. Błaszczyk, M. Stępień, S. Szabowski, W. Krysiak, M. Szymańska, Tarnów: J. Karasiński, J. Blicharz, M. Skura, Warsaw: K. Hałas, L. Michalczyk, Z. Orski, K. Krzyżanowski, A. Skrobowski, Warsaw: L. Zieliński, M. Tomaszewska-Kiecana, M. Dłużniewski, Warsaw: M. Kiliszek, M. Peller, M. Budnik, P. Balsam, G. Opolski, A. Tymińska, K. Ozierański, A. Wancerz, Warsaw: A. Borowiec, E. Majos, R. Dabrowski, H. Szwed, Zabrze: A. Musialik-Lydka, Zabrze: A. Leopold-Jadczyk, E. Jedrzejczyk-Patej, M. Koziel, R. Lenarczyk, M. Mazurek, Z. Kalarus, Zabrze: K. Krzemien-Wolska, P. Starosta, E. Nowalany-Kozielska, Zakopane: A. Orzechowska, M. Szpot, M. Staszel, **PORTUGAL** Almada: S. Almeida, H. Pereira, L. Brandão Alves, R. Miranda, L. Ribeiro, Carnaxide Lisboa: F. Costa, F. Morgado, P. Carmo, P. Galvao Santos, R. Bernardo, P. Adragão, Santarém: G. Ferreira da Silva, M. Peres, M. Alves, M. Leal, Vila Real: A. Cordeiro, P. Magalhães, P. Fontes, S. Leão, Viseu: A. Delgado, A. Costa, B. Marmelo, B. Rodrigues, D. Moreira, J. Santos, L. Santos, **ROMANIA** Arad: A. Terchet, D. Darabantiu, S. Mercea, V. Turcin Halka, A. Pop Moldovan, Brasov: A. Gabor, B. Doka, G. Catanescu, H. Rus, L. Oboroceanu, E. Bobescu, Bucharest: R. Popescu, A. Dan, A. Buzea, I. Daha, G. Dan, I. Neuhoff, Bucharest: M. Baluta, R. Ploesteanu, N. Dumitrache, M. Vintila, Bucharest: A. Daraban, C. Japie, E. Badila, H. Tewelde, M. Hostiuc, S. Frunza, E. Tintea, D. Bartos, Bucharest: A. Ciobanu, I. Popescu, N. Toma, C. Gherghinescu, D. Cretu, N. Patrascu, C. Stoicescu, C. Udroiu, G. Bicescu, V. Vintila, D. Vinereanu, M. Cinteza, R. Rimbas, Iași: M. Grecu, Oradea: A. Cozma, F. Boros, M. Ille, O. Tica, R. Tor, A. Corina, A. Jeewooth, B. Maria, C. Georgiana, C. Natalia, D. Alin, D. Dinu-Andrei, M. Livia, R. Daniela, R. Larisa, S. Umaar, T. Tamara, M. Ioachim Popescu, Târgu Mureș: D. Nistor, I. Sus, O. Coborosanu, Timișoara: N. Alina-Ramona, R. Dan, L. Petrescu, Timișoara: G. Ionescu, I. Popescu, C. Vacarescu, E. Goanta, M. Mangea, A. Ionac, C. Mornos, D. Cozma, S. Pescariu, **RUSSIAN FEDERATION** Arkhangelsk: E. Solodovnicova, I. Soldatova, J. Shutova, L. Tjuleneva, T. Zubova, V. Uskov, Arkhangelsk: D. Obukhov, G. Rusanova, Arkhangelsk: I. Soldatova, N. Isakova, S. Odinsova, T. Arhipova, Arkhangelsk: E. Kazakevich, E. Serdechnaya, O. Zavyalova, Saint-Petersburg: T. Novikova, Saint-Petersburg: I. Riabaia, S. Zhigalov, Saint-Petersburg: E. Drozdova, I. Luchkina, Y. Monogarova, Vladivostok: D. Hegya, L. Rodionova, L. Rodionova, V. Nevzorova, Vladivostok: I. Soldatova, O. Lusanova, **SERBIA** Belgrade: A. Arandjelovic, D. Toncev, M. Milanov, N. Sekularac, Belgrade: M. Zdravkovic, S. Hinic, S. Dimkovic, T. Acimovic, J. Saric, Belgrade: M. Polovina, T. Potpara, B. Vujisic-Tesic, M. Nedeljkovic, Belgrade: M. Zlatar, M. Asanin, Belgrade: V. Vasic, Z. Popovic, Belgrade: D. Djikic, M. Sipic, V. Peric, B. Dejanovic, N. Milosevic, Belgrade: A. Stevanovic, A. Andric, B. Pencic, M. Pavlovic-Kleut, V. Celic, Kragujevac: M. Pavlovic, M. Petrovic, M. Vuleta, N. Petrovic, S. Simovic, Z. Savovic, S. Milanov, G. Davidovic, V. Iric-Cupic, Niška Banja: D. Simonovic, M. Stojanovic, S. Stojanovic, V. Mitic, V. Ilic, D. Petrovic, M. Deljanin Ilic, S. Ilic, V. Stoickov, Pirot: S. Markovic, Šabac:S. Kovacevic. **SPAIN** Alicante: A. García Fernandez, Benalmadena: A. Perez Cabeza, Córdoba: M. Anguita, Granada: L. Tercedor Sanchez, Huarte: E. Mau, J. Loayssa, M. Ayarra, M. Carpintero, Madrid: I. Roldán Rabadan, Murcia: M. Leal, Murcia: M. Gil Ortega, Murcia: A. Tello Montoliu, E. Orenes Piñero, S. Manzano Fernández, F. Marín, A. Romero Aniorte, A. Veliz Martínez, M. Quintana Giner, Pamplona: G. Ballesteros, M. Palacio, O. Alcalde, I. García-Bolao, San Juan de Alicante: V. Bertomeu Gonzalez, Santiago de Compostela: F. Otero-Raviña, J. García Seara, J. Gonzalez Juanatey, **SWITZERLAND** Geneva: N. Dayal, P. Maziarski, P. Gentil-Baron, D. Shah, **TURKEY** Adana: M. Koç, Afyon: E. Onrat, I. E. Dural, Ankara: K. Yilmaz, B. Özin, Ankara: S. Tan Kurklu, Y. Atmaca, Ankara: U. Canpolat, L. Tokgozoglu, Ankara: A. K. Dolu, B. Demirtas, D. Sahin, Ankara: O. Ozcan Celebi, E. Diker, Antalya: G. Gagirci, Bayraklı/Izmir: U.O.Turk, Bursa: H. Ari, Diyarbakır: N. Polat, N. Toprak, Gaziantep: M. Sucu, Görükle-Bursa: O. Akin Serdar, Istanbul: A. Taha Alper, Istanbul: A. Kepez, Istanbul: Y. Yuksel, Kurupelit - Samsun: A. Uzunselvi, S. Yuksel, M. Sahin, Merkez/Düzce: O. Kayapinar, Mersin: T. Ozcan, Sivas: H. Kaya, M. B. Yilmaz, Trabzon: M. Kutlu, Yüreğir-Adana: M. Demir, **UNITED KINGDOM** Barnstaple: C. Gibbs, S. Kaminskiene, M. Bryce, A. Skinner, G. Belcher, J. Hunt, L. Stancombe, B. Holbrook, C. Peters, S. Tettersell, Birmingham: A. Shantsila, D. Lane, K. Senoo, M. Proietti, K. Russell, P. Domingos, S. Hussain, J. Partridge, R. Haynes, S. Bahadur, R. Brown, S. McMahon, G. Y H Lip, Blackburn: J. McDonald, K. Balachandran, R. Singh, S. Garg, H. Desai, K. Davies, W. Goddard, Blackpool: G. Galasko, I. Rahman, Y. Chua, O. Payne, S. Preston, O. Brennan, L. Pedley, C. Whiteside, C. Dickinson, J. Brown, K. Jones, L. Benham, R. Brady, Carlisle: L. Buchanan, A. Ashton, H. Crowther, H. Fairlamb, S. Thornthwaite, C. Relph, A. McSkeane, U. Poultney, N. Kelsall, P. Rice, T. Wilson, Chertsey: M. Wrigley, R. Kaba, T. Patel, E. Young, J. Law, Cramlington: C. Runnett, H. Thomas, H. McKie, J. Fuller, S. Pick, Exeter: A. Sharp, A. Hunt, K. Thorpe, C. Hardman, E. Cusack, L. Adams, M. Hough, S. Keenan, A. Bowring, J. Watts, Great Yarmouth: J. Zaman, K. Goffin, H. Nutt, Harrogate: Y. Beerachee, J. Featherstone, C. Mills, J. Pearson, L. Stephenson, Huddersfield: S. Grant, A. Wilson, C. Hawksworth, I. Alam, M. Robinson, S. Ryan, Macclesfield: R. Egdell, E. Gibson, M. Holland, D. Leonard, Maidstone: B. Mishra, S. Ahmad, H. Randall, J. Hill, L. Reid, M. George, S. McKinley, L. Brockway, W. Milligan, Manchester: J. Sobolewska, J. Muir, L. Tuckis, L. Winstanley, P. Jacob, S. Kaye, L. Morby, Nottingham: A. Jan, T. Sewell, Poole: C. Boos, B. Wadams, C. Cope, P. Jefferey, Portsmouth: N. Andrews, A. Getty, A. Suttling, C. Turner, K. Hudson, R. Austin, S. Howe, Redhill: R. Iqbal, N. Gandhi, K. Brophy, P. Mirza, E. Willard, S. Collins, N. Ndlovu, Rhyl: E. Subkovas, V. Karthikeyan, L. Waggett, A. Wood, A. Bolger, J. Stockport, L. Evans, E. Harman, J. Starling, L. Williams, V. Saul, Salisbury: M. Sinha, L. Bell, S. Tudgay, S. Kemp, J. Brown, L. Frost, Shrewsbury: T. Ingram, A. Loughlin, C. Adams, M. Adams, F. Hurford, C. Owen, C. Miller, D. Donaldson, H. Tivenan, H. Button, South Shields: A. Nasser, O. Jhagra, B. Stidolph, C. Brown, C. Livingstone, M. Duffy, P. Madgwick, Southampton: P. Roberts, E. Greenwood, L. Fletcher, M. Beveridge, S. Earles, Taunton: D. McKenzie, D. Beacock, M. Dayer, M. Seddon, D. Greenwell, F. Luxton, F. Venn, H. Mills, J. Rewbury, K. James, K. Roberts, L. Tonks, Torquay: D. Felmeden, W. Taggu, A. Summerhayes, D. Hughes, J. Sutton, L. Felmeden, Watford: M. Khan, E. Walker, L. Norris, L. O'Donohoe, Weston-super-Mare: A. Mozid, H. Dymond, H. Lloyd-Jones, G. Saunders, D. Simmons, D. Coles, D. Cotterill, S. Beech, S. Kidd, Wolverhampton: B. Wrigley, S. Petkar, A. Smallwood, R. Jones, E. Radford, S. Milgate, S. Metherell, V. Cottam, Yeovil: C. Buckley, A. Broadley, D. Wood, J. Allison, K. Rennie, L. Balian, L. Howard, L. Pippard, S. Board, T. Pitt-Kerby.
